# Supplementary material for: Pooled CRISPR interference screening enables genome-scale functional genomics study in bacteria with superior performance
Source: Nat Commun. 2018 Jun 26;9:2475. doi: 10.1038/s41467-018-04899-x (PMC6018678; doi:10.1038/s41467-018-04899-x)
Supplement: Supplementary file 1 — Supplementary Information [file 41467_2018_4899_MOESM1_ESM.pdf]

Pooled CRISPR interference screening enables genome-scale functional genomics study in bacteria with superior performance Wang et al.

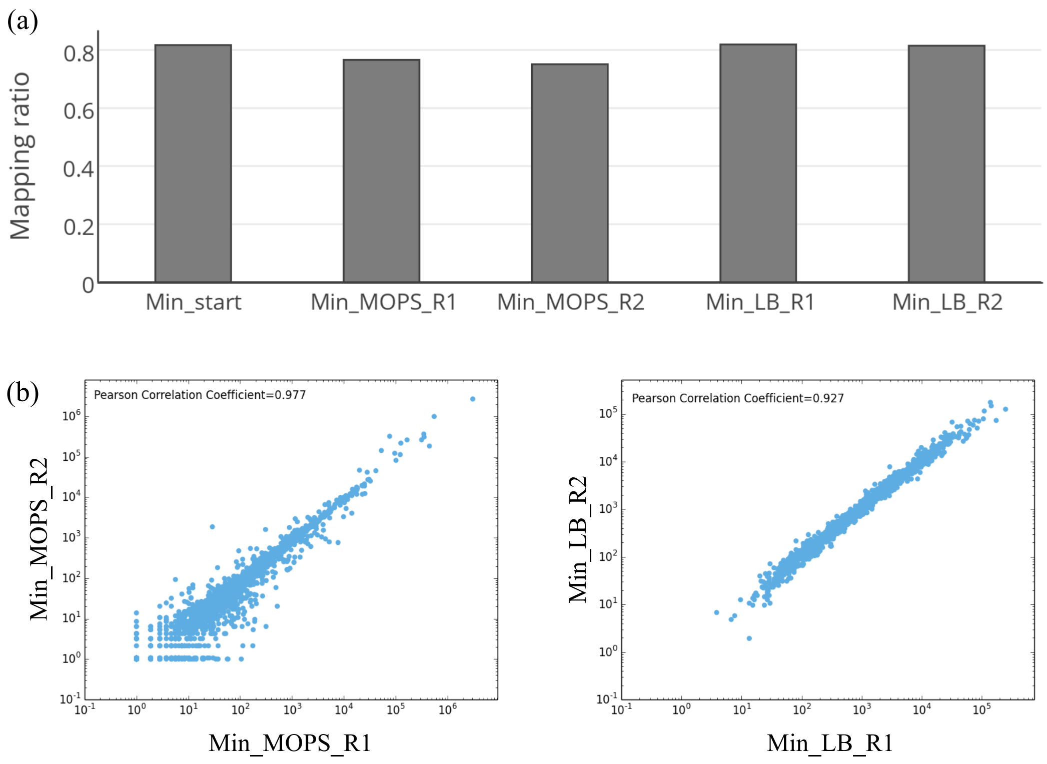

Supplementary Fig. 1 Quality of tiling library-screening experiments. (a) Mapping ratios of five NGS libraries constructed in tiling library screening experiments. 'Min_Start' refers to the initial library in plasmid format; R1 and R2 refer to biological replicates. (b) Biological replicates of tiling library screenings give very consistent results. Normalized read number is plotted for each sgRNA.

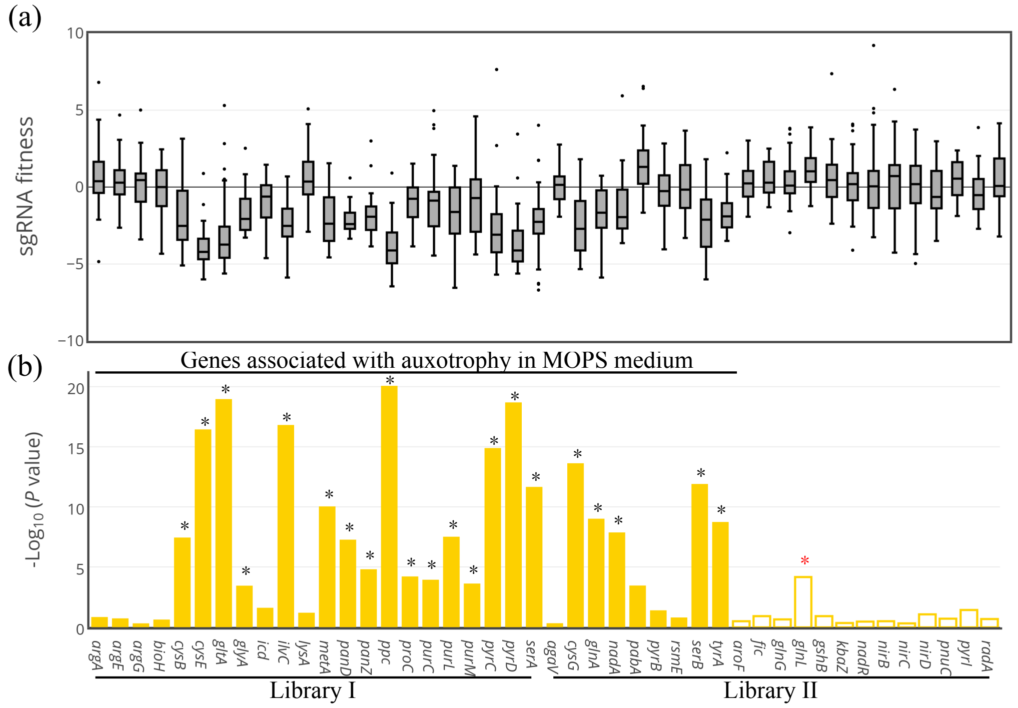

Supplementary Fig. 2 Genes that are auxotrophic in MOPS media can be robustly recovered by CRISPRi-based pooled screen. (a) Box plot of sgRNA fitness score distributions for genes in Libraries I and II. Outliers outside the 1.5-fold interquartile range from the box boundary were plotted individually (dots). For clarity, only sgRNAs with fitness scores between –10 and 10 were plotted. (b) $-\text{Log}_{10}$(*P* value), where *P* values were derived from two-tailed Mann-Whitney U tests of sgRNA fitness scores belonging to the indicated gene against the control sgRNA set. Filled bars represent genes with auxotrophic phenotypes reported in the Keio collection characterization; unfilled bars represent genes in Library II for which knockout does not impair growth in MOPS. Asterisks indicate *P* value by two-tailed Mann Whitney U test < 0.01; red asterisks indicate false positives with significant *P* values, but belonging to the non-auxotrophic group

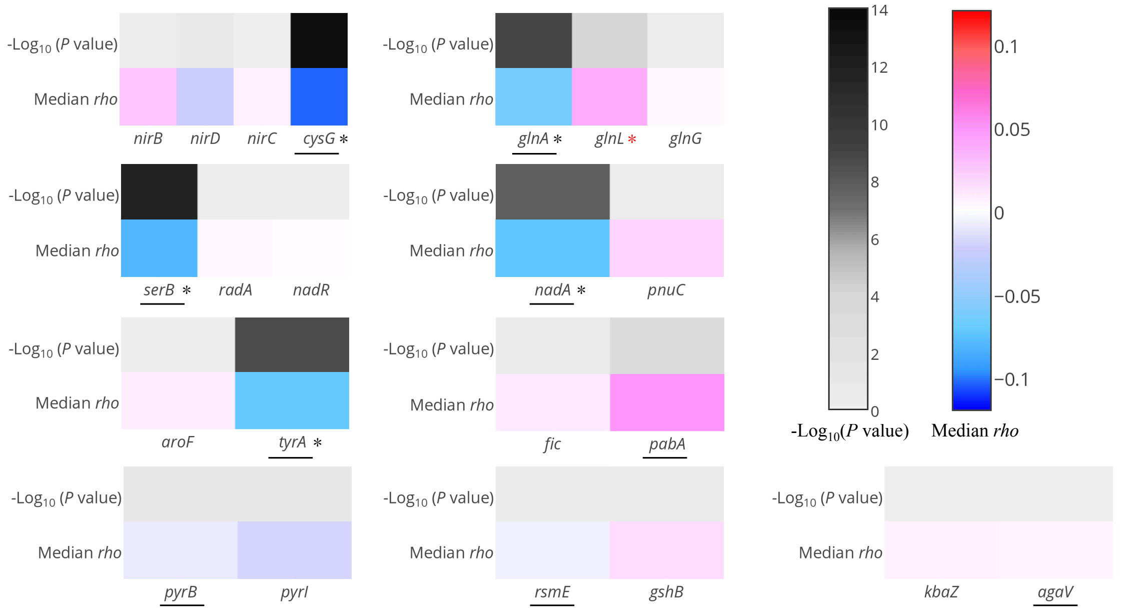

Supplementary Fig. 3 CRISPRi-based pooled screening (tiling library experiment) yields robust identification of phenotype-associated genes in polycistronic-mRNA-transcribing operons. Heat maps of –Log$_{10}$(*P* value) and median sgRNA fitness score are shown for all nine polycistronic operons in Library II. Auxotrophic genes in MOPS media are underlined; significant hits (*P* value by two-tailed MWU test < 0.01) are indicated with asterisks. Black asterisks are true positive; red asterisks indicate false positives with significant *P* values, but belonging to the non-auxotrophic group.

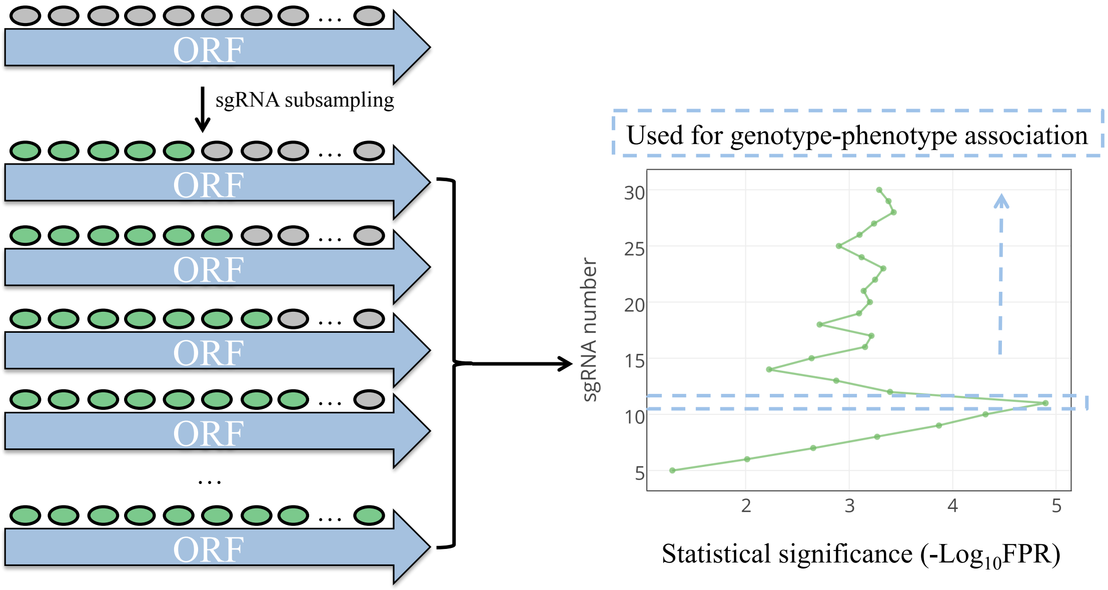

Supplementary Fig. 4 Schematic workflow of optimized hit-gene calling algorithm. sgRNA subsets are collected from the sgRNA pool of one gene (gray circles, top arrow). The initial subsample consists of the five sgRNAs (if available) most proximal to the start codon (green circles, second arrow). Then, one sgRNA most proximal to the start codon in the remaining set (green circles) is added per cycle. For each sgRNA subset, statistical tests are performed to calculate a FPR value (see Methods) for this gene. The set with the strongest statistical significance (smallest FPR value) is used for genotype-phenotype association metric calculation.

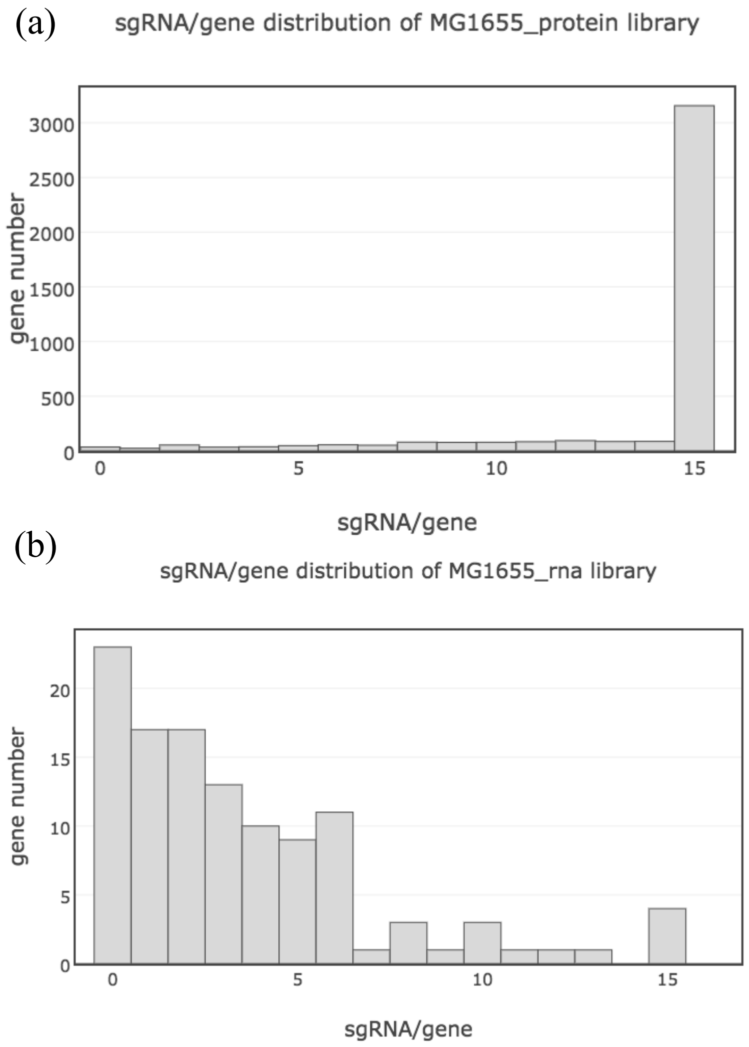

Supplementary Fig. 5 Histogram of sgRNA number designed for each gene in *E. coli* genome-scale sgRNA library. (a) Protein-coding genes, (b) ncRNA-coding genes.

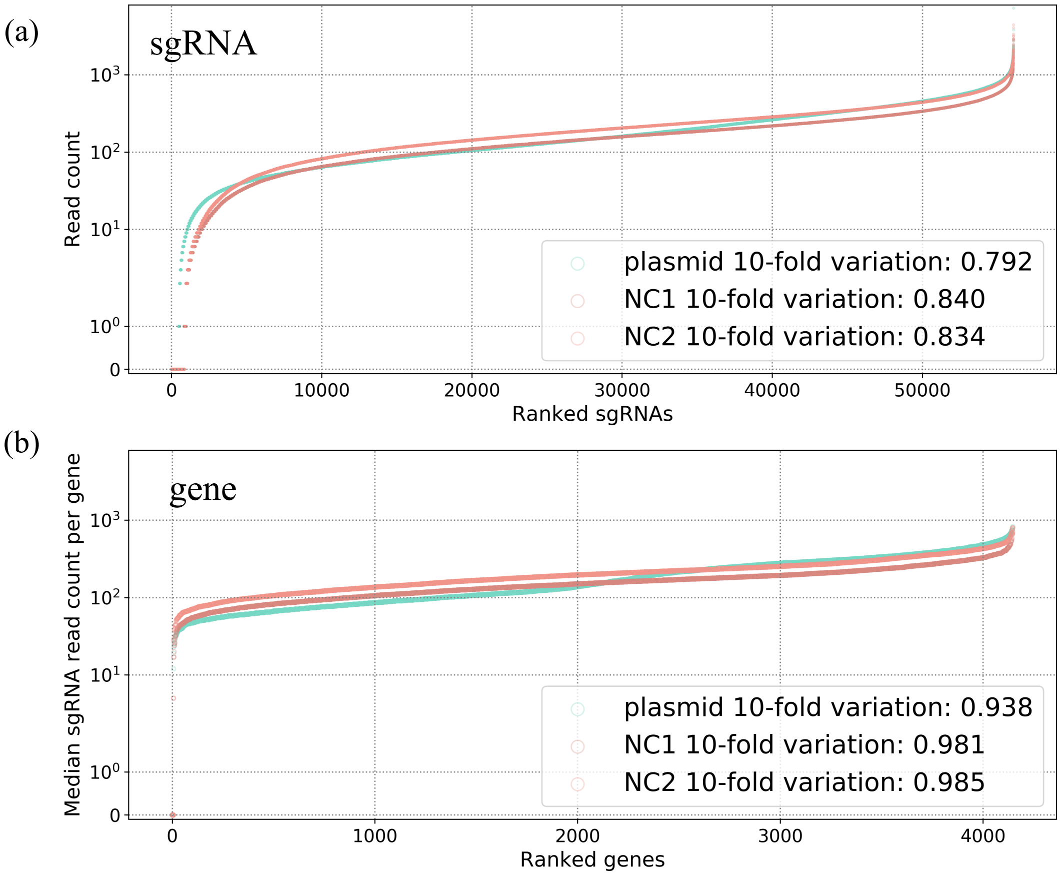

Supplementary Fig. 6 NGS profiling of *E. coli* genome-wide sgRNA library in plasmid format (green, plasmid) or after transformation and overnight cultivation (red, NC1 and NC2). (a) Library profile at sgRNA level presented by read count distribution of sgRNAs detected in NGS; (b) Library profile at gene level by median sgRNA read count (for each gene) distribution detected in NGS. 10-fold variation is shown for all distribution profiles calculated by summarizing the event number (sgRNA or gene) with read count belonging to [1/3.33×median event read count, 3.33×median event read count], and normalized by the total event number.

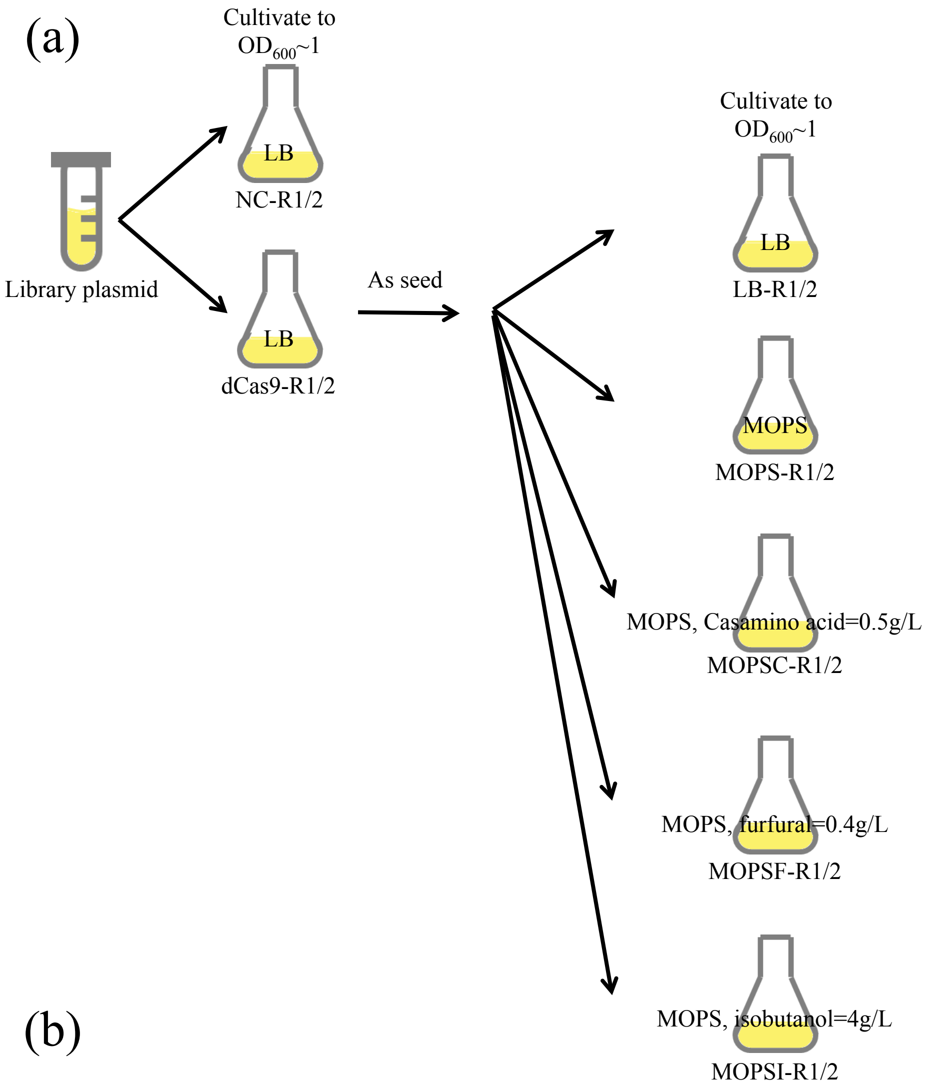

(b)

Phenotype	Selective condition	Control condition	Initial condition
Essentiality	dCas9-R1/2	NC-R1/2	Library plasmid
Auxotrophy	MOPS-R1/2	LB-R1/2	initial#
L-Trp biosynthesis	MOPSC-R1/2	LB-R1/2	initial
Furfural tolerance	MOPSF-R1/2	initial	initial
Isobutanol tolerance	MOPSI-R1/2	initial	initial

mixture of dCas9-R1/2

Supplementary Fig. 7 Workflow (a) and phenotype studied (b) in CRISPRi screening of the *E. coli* genome-wide sgRNA library.

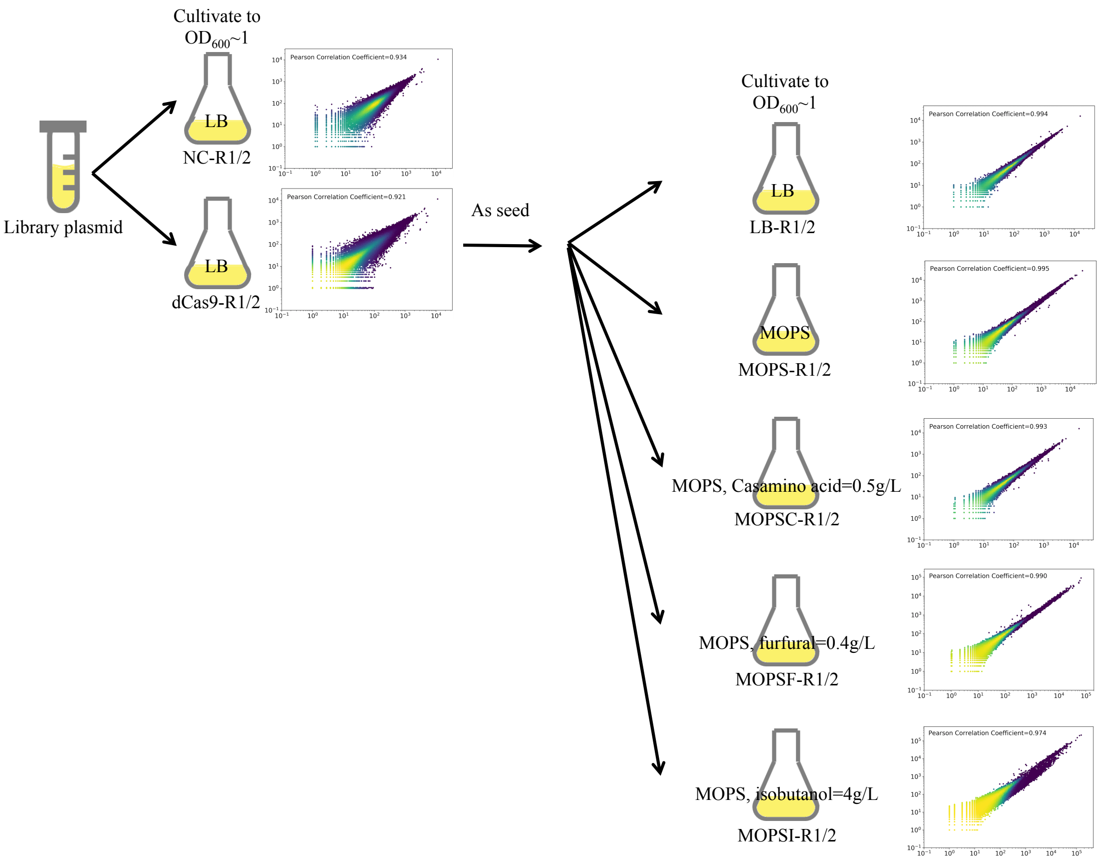

Supplementary Fig. 8 Biological replicates of genome-wide sgRNA library screenings give very consistent results. Normalized read count number is plotted for each sgRNA. Pearson correlation coefficient: 0.934 (NC-R1/2), 0.921 (dCas9-R1/2), 0.994 (LB-R1/2), 0.995 (MOPS-R1/2), 0.993 (MOPSC-R1/2), 0.990 (MOPSF-R1/2) and 0.974 (MOPSI-R1/2).

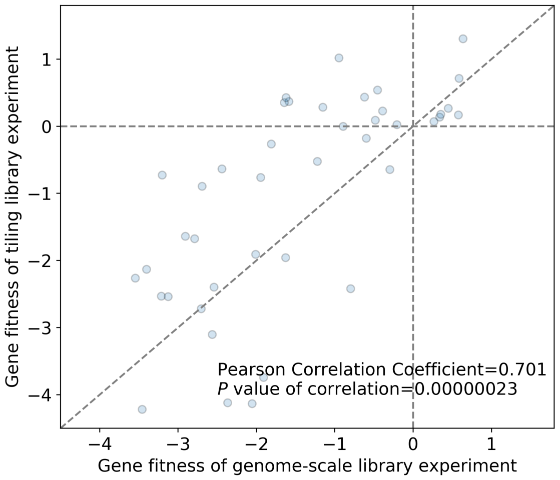

Supplementary Fig. 9 Comparison between gene fitness score for auxotrophy phenotype obtained from genome-scale library screening (X axis) and tiling library screening (Y axis). In spite of good agreement between these two groups of results, it should be noted that these two experiments are performed with different parameter settings, which might be the main source of the moderate difference. We experimentally confirmed that metabolite cross-feeding in pooled screening resulted in failure to identify the auxotrophic genes (for example, *argA*, *argE* and *argG* are found to be false negative in tiling library screening, see Supplementary Fig. 2) by cultivating auxotrophic-gene-knockdown mutant in 'cultivated' filter-sterilized MOPS media supplemented with fresh carbon source and measuring growth (for this experiment, see our post on bioRxiv, doi: 10.1101/129668). This failure mode is more significant with prolonged pooled screening time (10 doublings in tiling library screening and 5 doublings in genome-scale library screening) due to the accumulation of the cross-feeding metabolites in the media. That is also the reason why we optimized our screening protocol (decrease the cultivation time) in genome-wide screening experiments (*argA*, *argE* and *argG* are found to be auxotrophic genes in genome-wide screening).

(a)

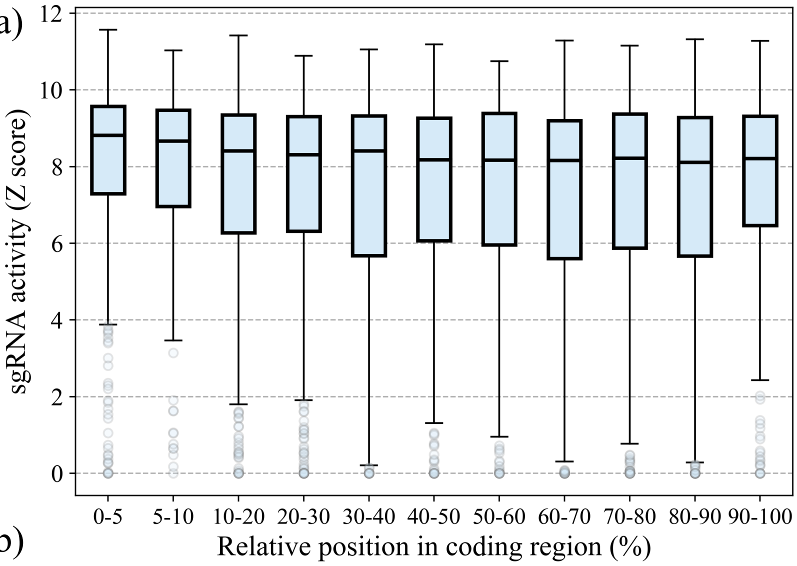

(b)

$-\text{Log}_{10}P$ MWU test	Position (% coding region)	sgRNA number
8.12	0-5	597
1.53	5-10	196
0.15	10-20	388
0.17	20-30	370
0.28	30-40	405
0.51	40-50	406
0.61	50-60	401
1.77	60-70	389
0.51	70-80	393
1.22	80-90	388
0.39	90-100	240

Supplementary Fig. 10 The data of genome-wide sgRNA activities adopted from gene essentiality screening experiments confirms that (a) sgRNAs residing within the first 5% of ORF region are significantly more active, supporting our observation in tiling library screening (Figure 1b). The absolute values of the *Z* scores for each functional sgRNA targeting the 337 true positive essential genes (Here we applied a threshold of FDR < 0.01, fitness < –4 on the dataset to extract true positive essential genes) are extracted and the distribution of each group (categorized via position in ORF) against all 4,173 sgRNAs is tested by two-tailed Mann-Whitney U test (b). Outliers outside the 1.5-fold interquartile range from the box boundary are plotted individually (dots).

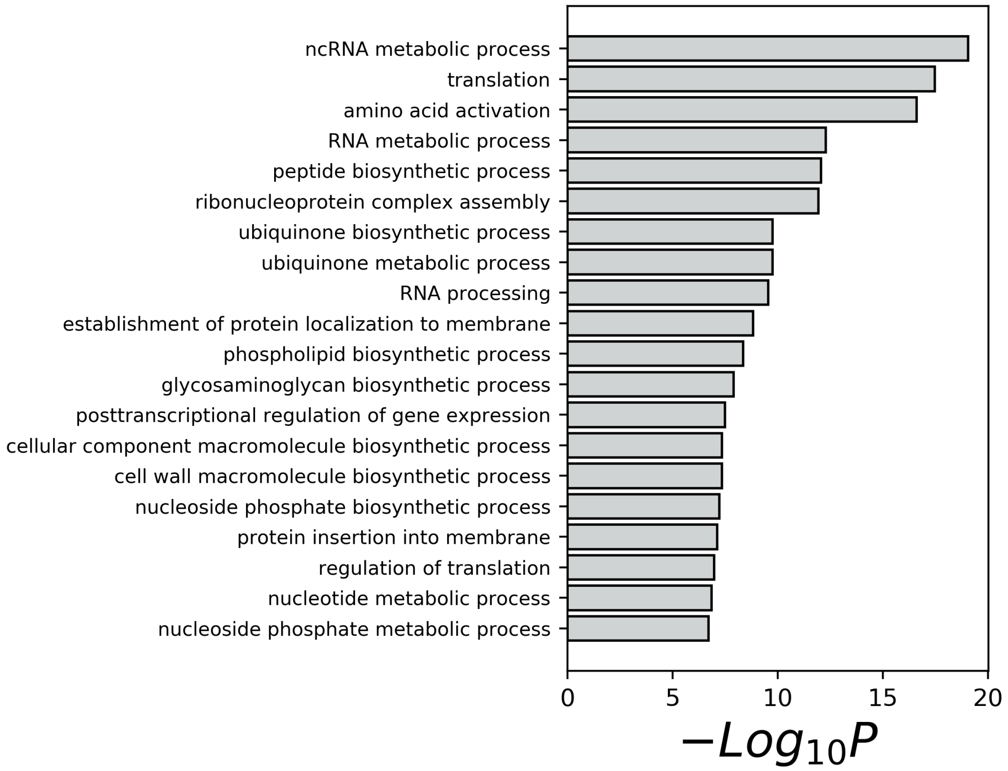

Supplementary Fig. 11 GO enrichment analysis of essential genes identified by CRISPRi screening in rich media (LB broth). *P* values are derived from two-tailed Fisher exact test.

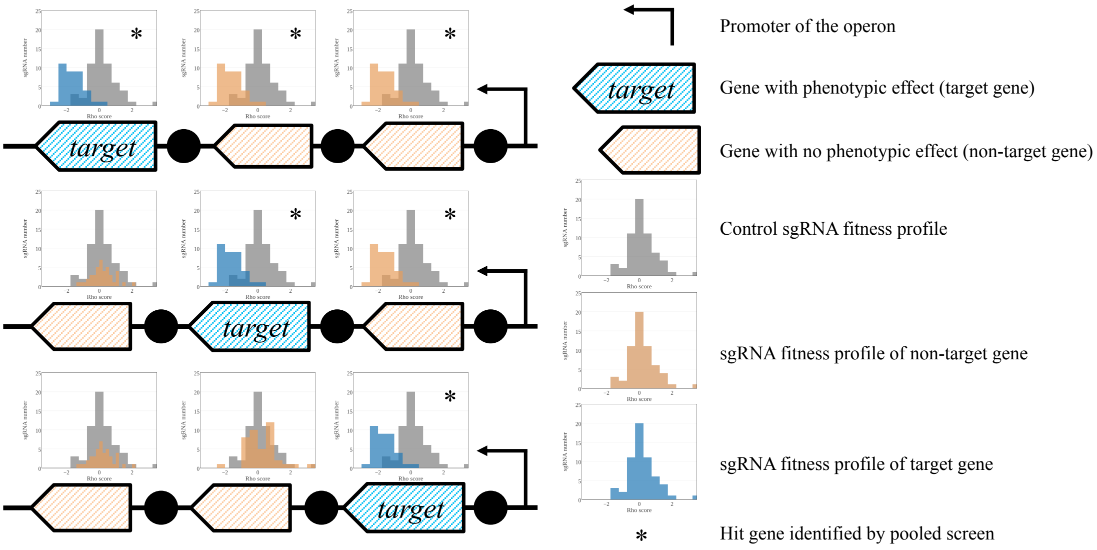

Supplementary Fig. 12 Rationale of gene fitness derived from CRISPRi screening when coming to genes organized in operons transcribed as polycistronic mRNA. Due to the working mechanism of CRISPRi complex as a roadblock to repress transcription, the transcription of genes downstream of the sgRNA target site should be repressed. Hence, (Upper panel) if the functional target gene (genes whose knockdown significantly perturb growth of the cell thus identified by the pooled screening, coded as blue) locates at the 3' downstream of the operon, sgRNA targeting the upstream non-target genes (coded as orange) should also give rise to the repression of target gene expression, thus resulting in all genes in this operon identified as 'hit gene' (coded by asterisk); (Middle panel) if the functional target gene locates at the middle of the operon, the upstream non-target genes should also be identified as 'hit gene' while the downstream non-target genes not; (Lower panel) if the functional target gene locates at the 5' upstream of the operon, only itself should be identified as 'hit gene' while all others downstream not.

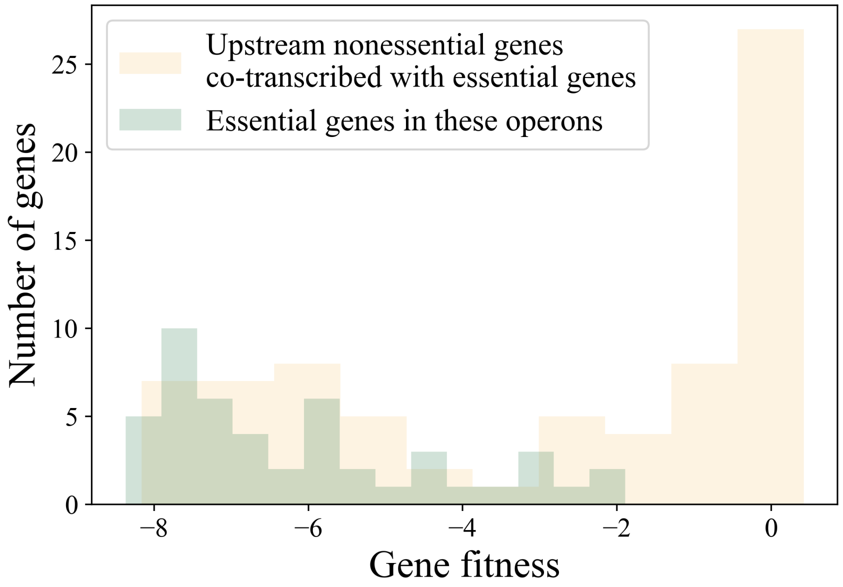

Supplementary Fig. 13 The forward polar effect of CRISPRi in polycistronic operons does not always hold true. Using all essential genes suggested by Keio collection with significant growth defect phenotype in CRISPRi screening (FDR < 0.05 and fitness < 0) as probe, we collected 47 polycistronic operons with such essential genes. The non-essential counterpart genes upstream of these probe essential genes in the same operon were extracted. In principle, according to Supplementary Fig. 11, the knockdown of these genes by CRISPRi should exhibit growth defect phenotype due to the forward polar effect. The histogram plots of gene fitness scores for these two categories of genes are presented (essential gene, green; upstream nonessential gene, yellow).

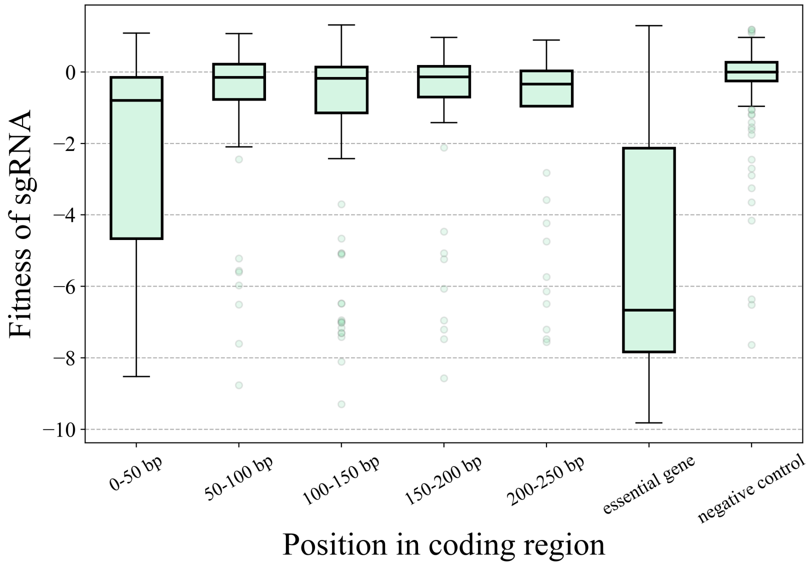

Supplementary Fig. 14 Position dependent reverse polarity of CRISPRi. All non-essential (according to Keio essential gene list) genes downstream of a known Keio essential gene in one common polycistronic operon were extracted, resulting in a set of 54 genes (663 sgRNAs). The hypothesis is that these genes exhibit no growth phenotype (average of sgRNAs), hence the phenotypes of some sgRNAs are derived from the reverse polarity. We observed strong phenotypes for sgRNAs within the first 50 bp in the coding regions of these genes (vs. negative control sgRNA: $P = 10^{-14.7}$; vs. all sgRNAs within 0-250 bp, $P = 10^{-3.3}$, two-tailed MWU test). As the distance from the upstream essential genes gets longer, such effect becomes weaker. Moreover, in spite of the reverse polarity within a 50 bp window, the phenotypes caused by such effect is significantly weaker than directly targeting dCas9-sgRNA complex to the essential genes ($P = 10^{-12.2}$, two-tailed MWU test), consistent with previous observations (Peters et al *Cell* 2016) that "forward" beats "reverse" in terms of strength. We also checked the overlaps between these 54 genes with their neighbor essential genes. We found only 3 of them have 3, 6 and 7 bp overlap with the upstream counterparts, thus ruling out the impact of overlaps on this analysis.

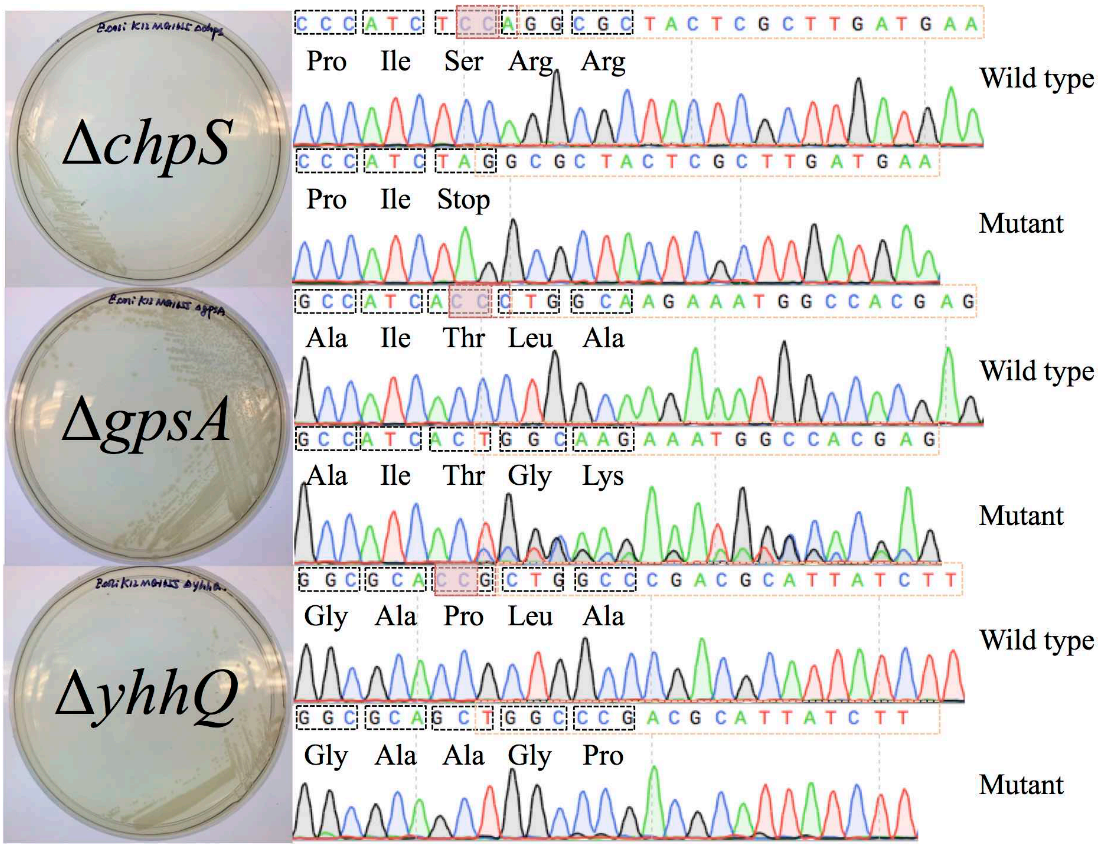

Supplementary Fig. 15 Frameshift mutations with 2 bp indel were successfully introduced into three genes. Shown on the left is the purified mutant strains on the LB agar plate. Right panel presents the Sanger sequencing result of wild type and mutant strain. Triple codons and relevant amino acids (or stop codon) are shown. Red rectangle represents PAM recognition site for CRISPR/Cas9 complex. Orange rectangle is the 20 nucleotides having Watson-Crick base pairing with sgRNAs. Guanine dimers (shown are is reverse complementary ‘CC’ at the non-template strand) within PAM that are deleted in mutant strain are highlighted by transparent light red.

Arg tRNAs

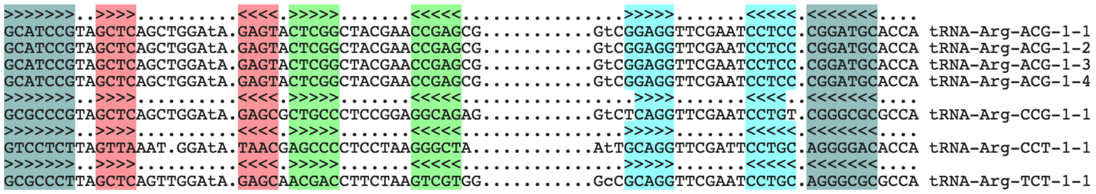

Gln tRNAs

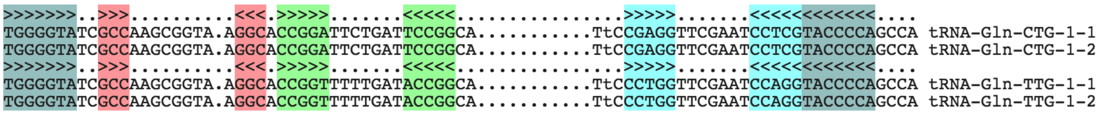

Gly tRNAs

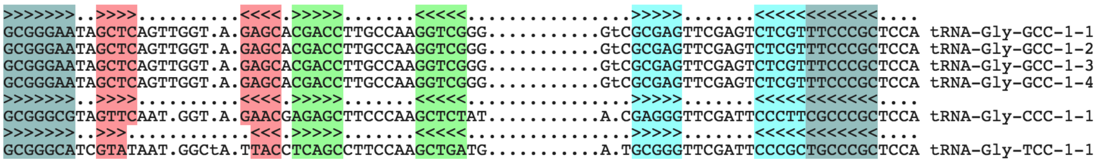

Supplementary Fig. 16 Representative tRNA isoacceptor families. The tRNA pool within a microorganism consists of various tRNA isoacceptor families (for example, tRNA-Arg-ACG). Each family has a different anticodon that decodes the corresponding codon. Each tRNA family has a single (e.g. tRNA-Arg-CCG) or multiple (e.g. tRNA-Arg-ACG) gene copies. In addition, due to the conserved structure of tRNA to fit the ribosome, all tRNAs encoded by a particular organism share overall sequence similarity (e.g. highlighted regions below in alignment). The figures are extracted from GtRNAdb and re-organized (Chan, P.P. & Lowe, T.M. (2016) GtRNAdb 2.0: an expanded database of transfer RNA genes identified in complete and draft genomes. Nucl. Acids Res. 44(Database issue):D184-D189.)

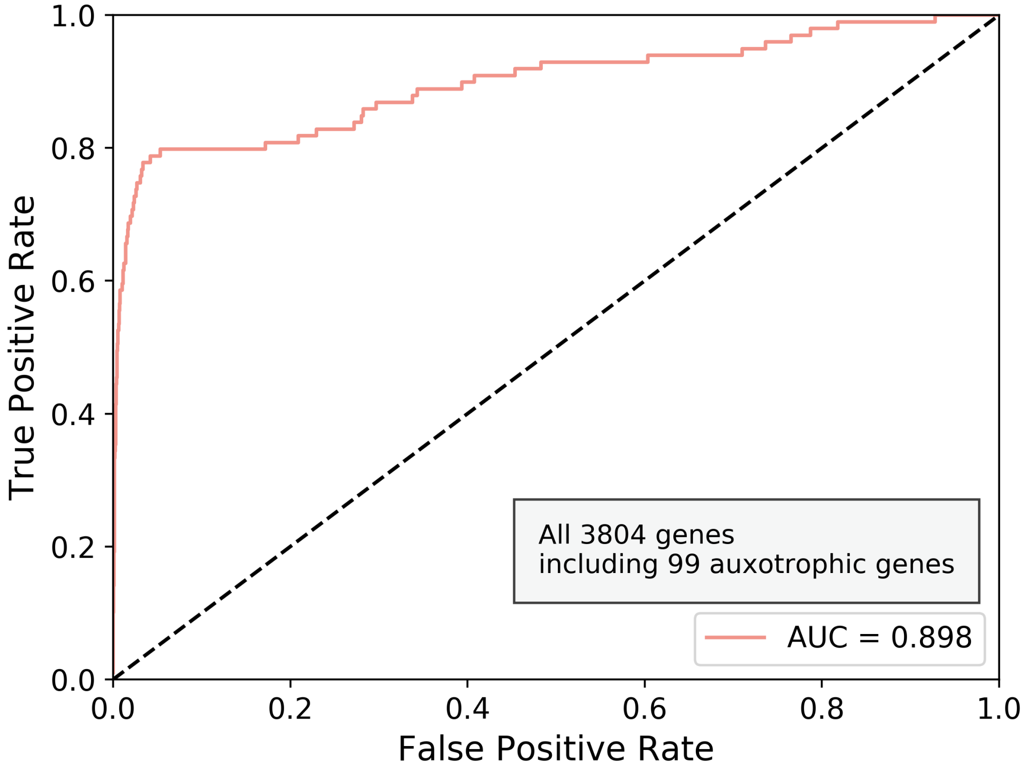

Supplementary Fig. 17 ROC curve presents the performance of CRISPRi screening in identifying 99 auxotrophic genes. Essential genes in rich media (FDR < 0.01, fitness < -4) are excluded from this analysis, resulting in 3,084 genes. True positive rates and false positive rates are calculated using a gold standard set of essential and nonessential genes by suggested by Nichols et al (*Cell* 2011). The dashed line represents the random guess of essential genes.

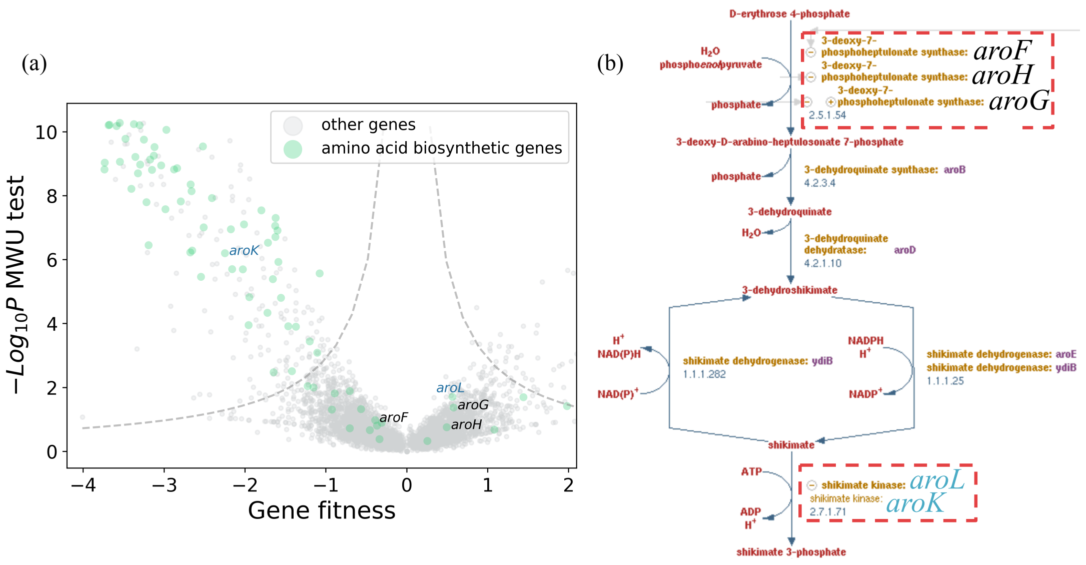

Supplementary Fig. 18 Fitness values in MOPS medium (a) of isoenzyme-coding genes in chorismate biosynthesis pathway (b). The biosynthesis pathway figure is adopted from the raw figure in EcoCyc.

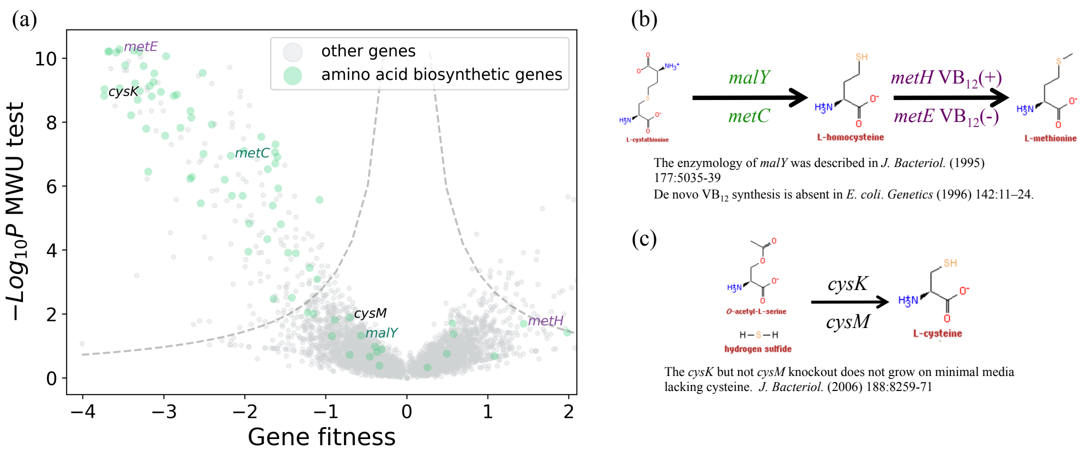

Supplementary Fig. 19 Fitness values in MOPS medium (a) of isoenzyme-coding genes in cysteine and methionine biosynthesis pathway (b). The biosynthesis pathway figure is adopted from the raw figure in EcoCyc.

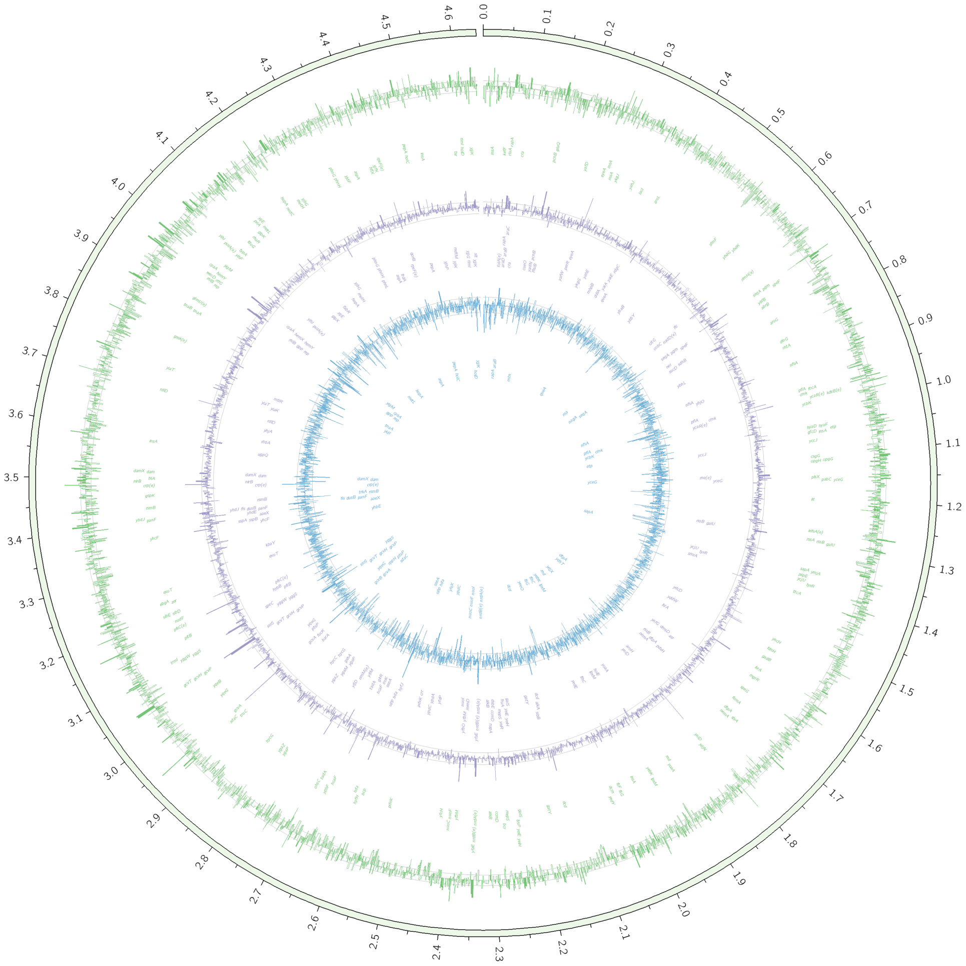

Supplementary Fig. 20 Genomic plots of fitness score of library variants in the presence of furfural (outer, green), isobutanol (middle, purple) and no supplementation (MOPS medium, inner, blue). The bars are relevant fitness values. All genes conferring significant (FDR < 0.05 for MOPS and furfural; FDR < 0.1 for isobutanol) tolerance phenotypes (positive fitness score) at these conditions are highlighted. Gene knockdowns with significant growth defect in rich medium (Figure 3a, fitness score < -4, FDR < 0.05) are labeled with '(e)' tag. For this figure with better resolution in .svg format, download at https://figshare.com/articles/Genome-scale_maps_for_toxic_chemical_tolerance/5513572 and view it by the web browser.

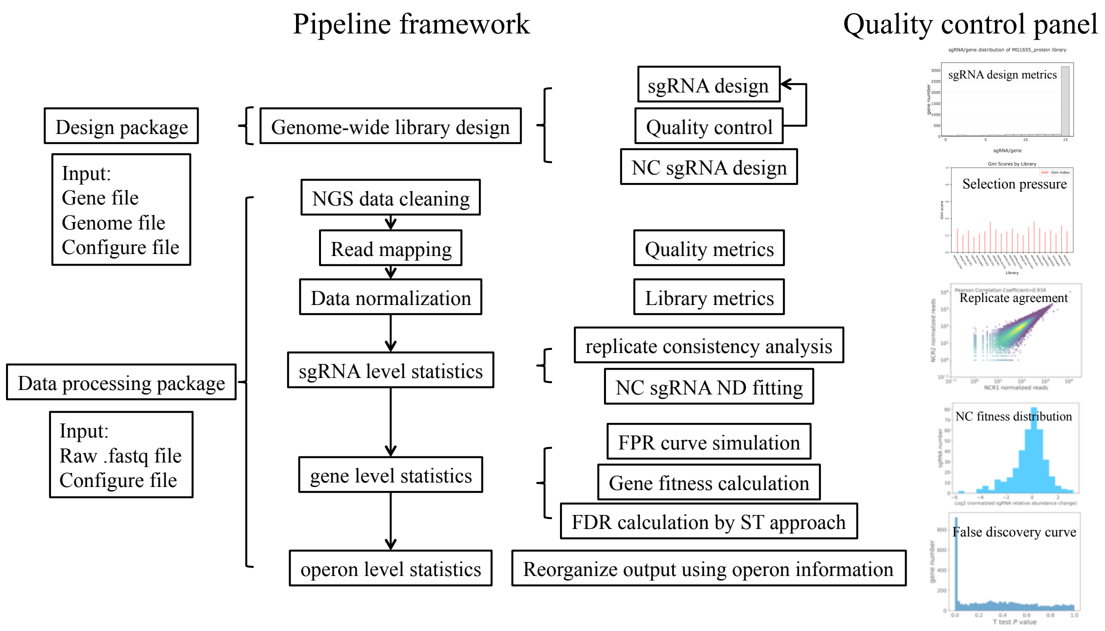

Supplementary Fig. 21 Framework and representative output figures of the software package developed in this work as a one-stop computational solution for the experimental biologists to use the CRISPRi screening method for prokaryotic functional genomics study. For the package and more details about utility, please check our GitHib site: https://github.com/zhangchonglab/CRISPRi-functional-genomics-in-prokaryotes

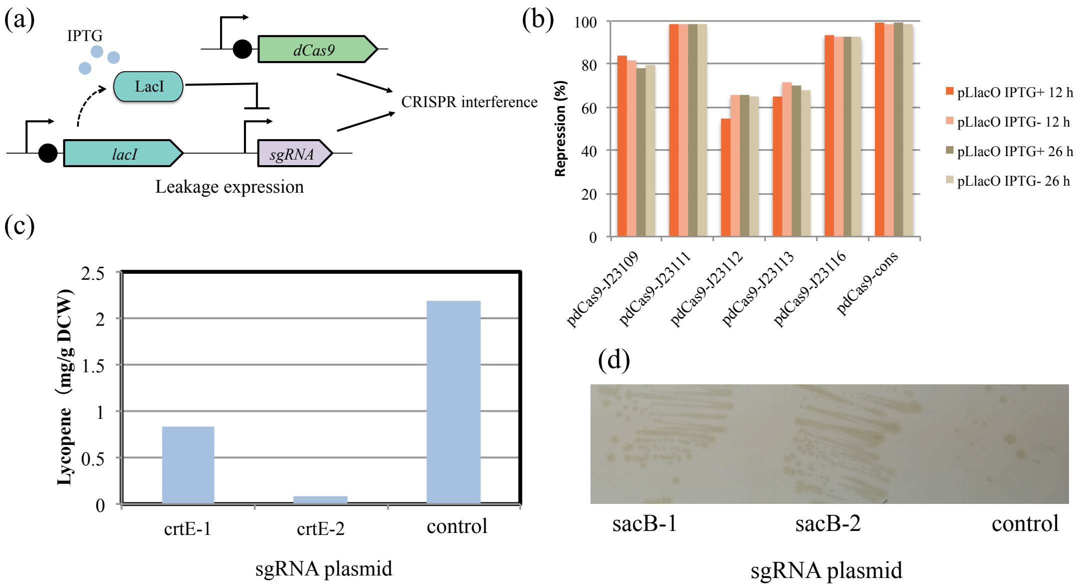

Supplementary Fig. 22 The CRISPRi system exhibited good repression of gene expression at the bulk population level. (a) Schematic illustration of CRISPRi system. (b) The CRISPRi system provided sustainable gene repression as measured by sfGFP fluorescence in a series of dCas9 constructs with diverse expression strength. (c) (d) shows the activity confirmation of the CRISPRi constructs used in this work to repress endogenous (integrated into the chromosome) gene expressions in *E. coli*. (c) The CRISPRi system targeting the lycopene biosynthesis pathway gene *crtE* via sgRNAs significantly reduced accumulation of lycopene in shaker flask fermentation. Plasmid pdCas9-J23111 was used. (d) Cellular toxicity of *sacB* in the presence of sucrose can be rescued by CRISPRi-induced gene repression (pdCas9-J23111).

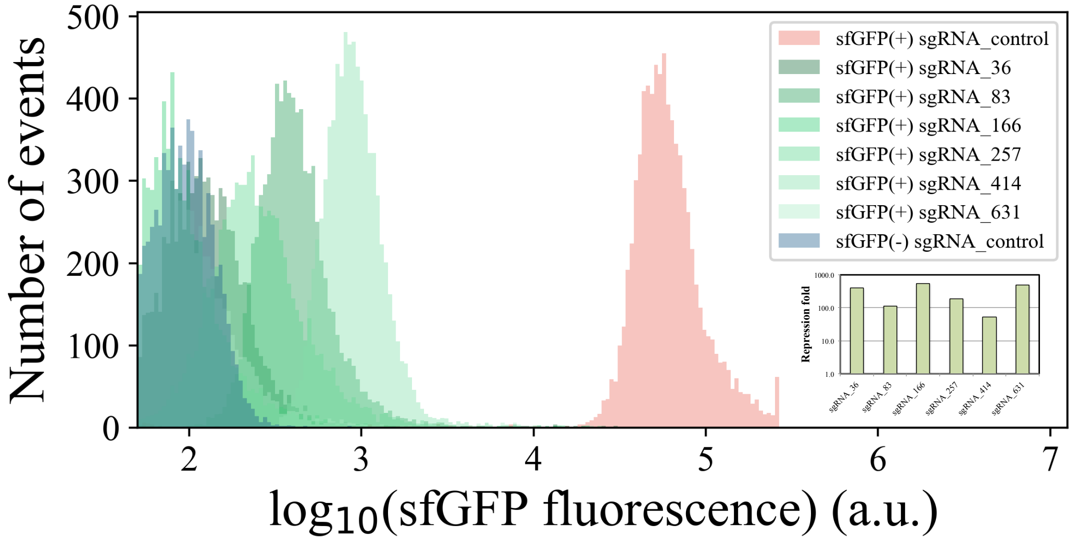

Supplementary Fig. 23 The CRISPRi system represses gene expression at the single-cell level. Shown is sfGFP expression repressed by different sgRNAs characterized by flow cytometry. Lower right presents the average repression fold for each sgRNA, sorted by their relative position in the sfGFP-coding region.

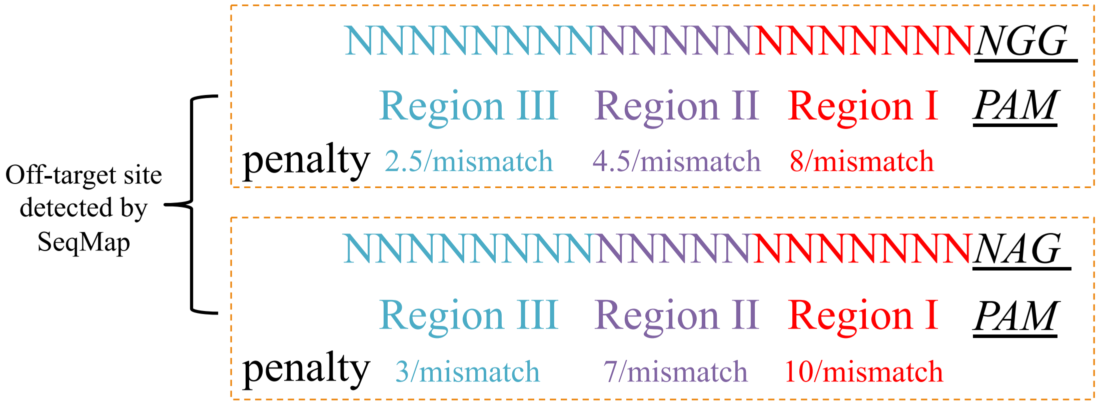

Σ(penalty×mismatch) > threshold: neglect off-target site

Σ(penalty×mismatch) < threshold: eliminate sgRNA candidate

Threshold = 11 for tiling library and 21 for genome-wide library

Supplementary Fig. 24 Scoring matrix used to measure the significance of sgRNA off-target hits. SeqMap was used to map the N20NG(A)G 23-mer to the *E. coli* genome. Identified potential off-target sites were scored by assigning different weights to mismatches at three regions of N20. The weighted sum of all mismatches was calculated and compared with a customized threshold (tiling library design, 11; genome-scale library design, 21). If the sum is smaller than the threshold, the off-target is considered significant and the corresponding sgRNA will be eliminated.

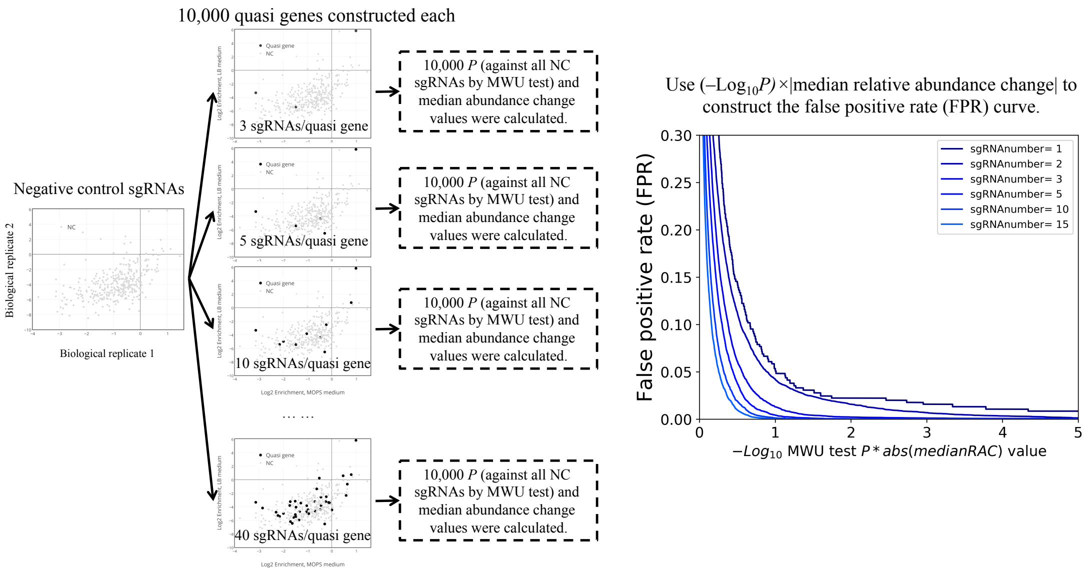

Supplementary Fig. 25 Schematic of method used in this work to construct FPR vs. score (|gene fitness| × -$\text{Log}_{10}(P)_{\text{MWU}}$) curve for genes with different numbers of sgRNAs via a 'quasi' gene simulation approach. For the detailed description of mathematics for this strategy, see Methods.

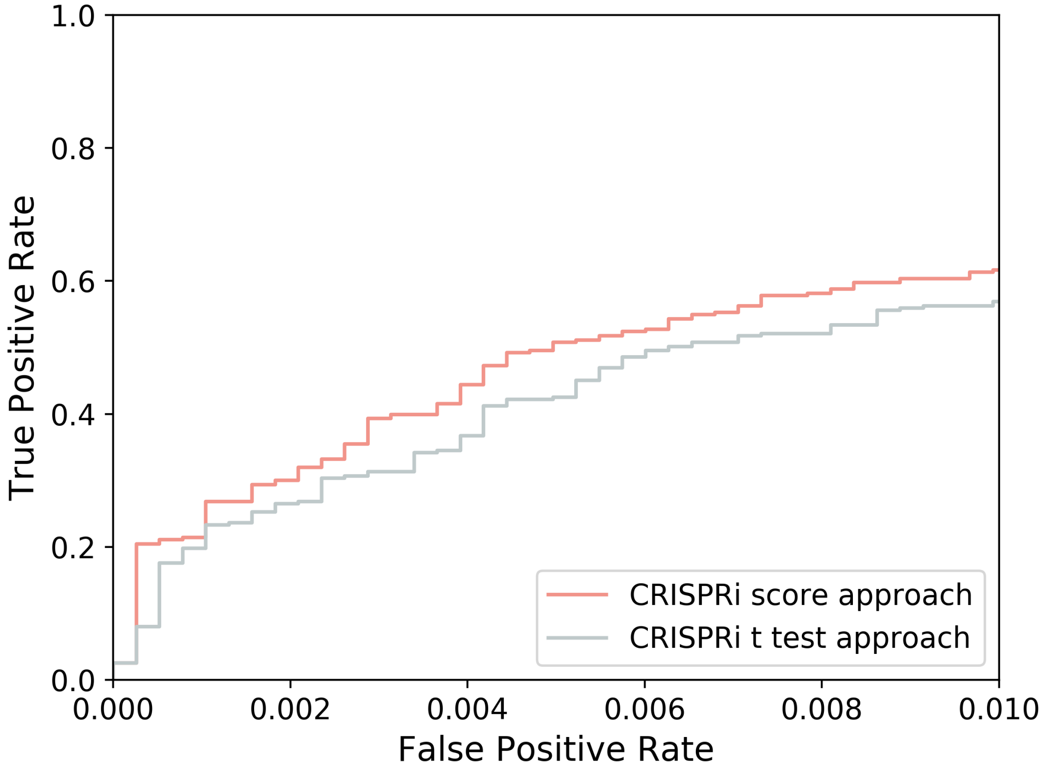

Supplementary Fig. 26 Comparison of different hit-gene calling methods (score method used in this work vs. t test method, for details, see Methods). FPR values obtained via these two methods are used to train a binary classifier to identify the essential genes. ROC curves indicate the performances of different methods in identifying essential genes when coping with all 4,140 protein-coding genes. True positive rates and false positive rates are calculated using a gold-standard set of essential and nonessential genes by Keio collection. Shown are ROC curves for score method (red, CRISPRi score) and t test method (grey, CRISPRi t test).

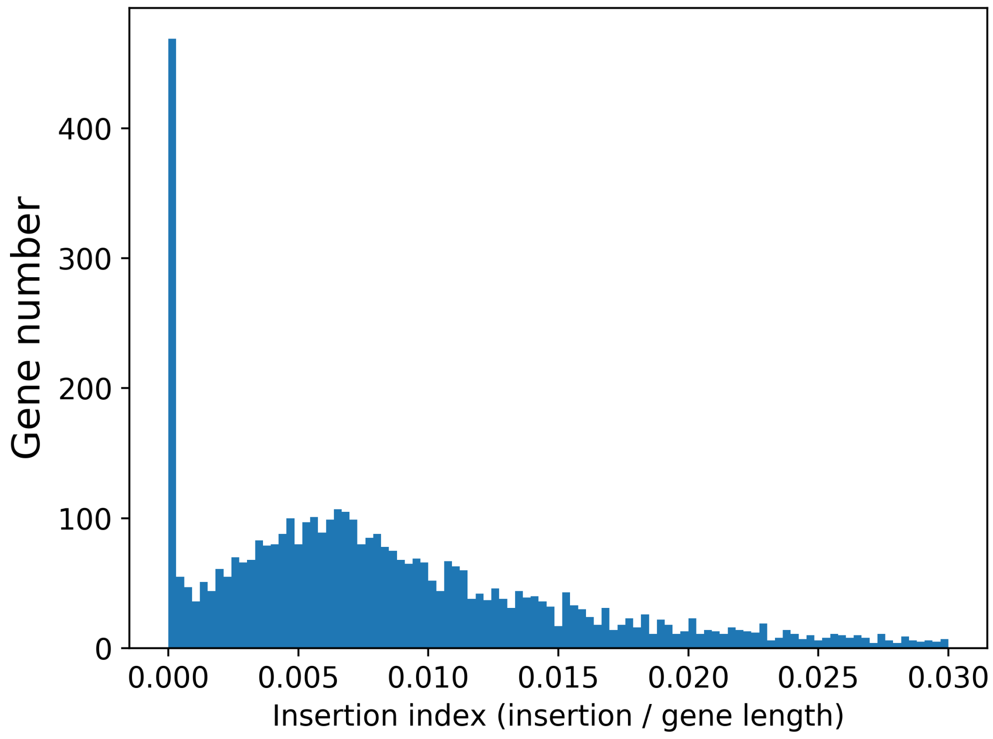

Supplementary Fig. 27 Selection of insertion index (number of unique transposon insertion events within the coding region normalized by the length of relevant genes) threshold to train an essential gene identification binary classifier by Tn-seq method. For details, see Methods.

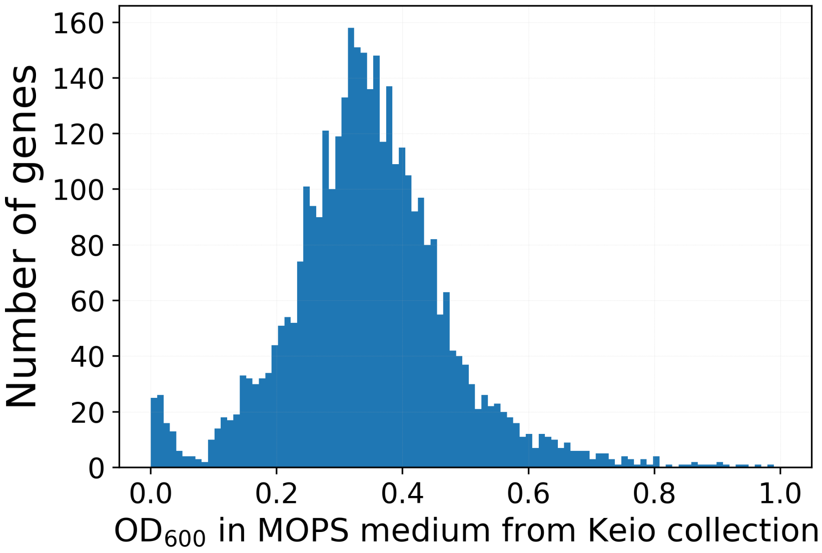

Supplementary Fig. 28 Selection of OD_{600} (from Keio dataset, 48 h cultivation in MOPS minimal medium) threshold to identify auxotrophic genes.

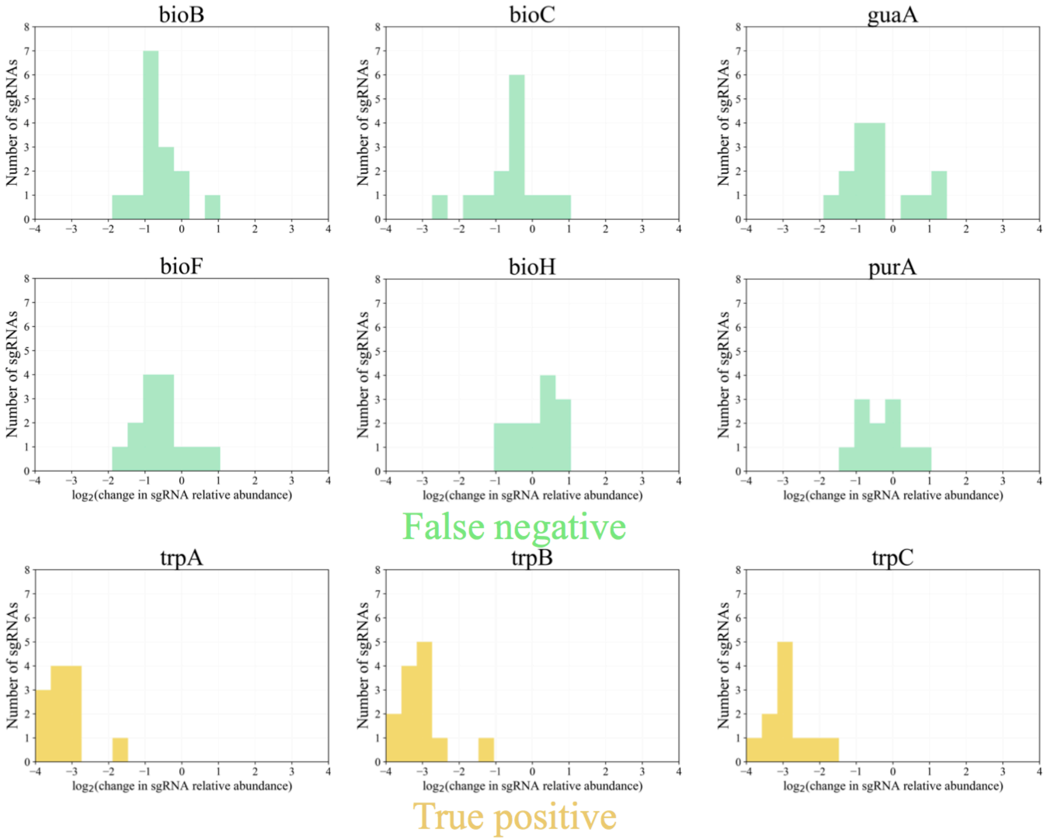

Supplementary Fig. 29 Six representative false negative auxotrophic genes (green) are selected; accompanied by three representative true positive genes (yellow). Their sgRNA fitness scores are shown as histogram.

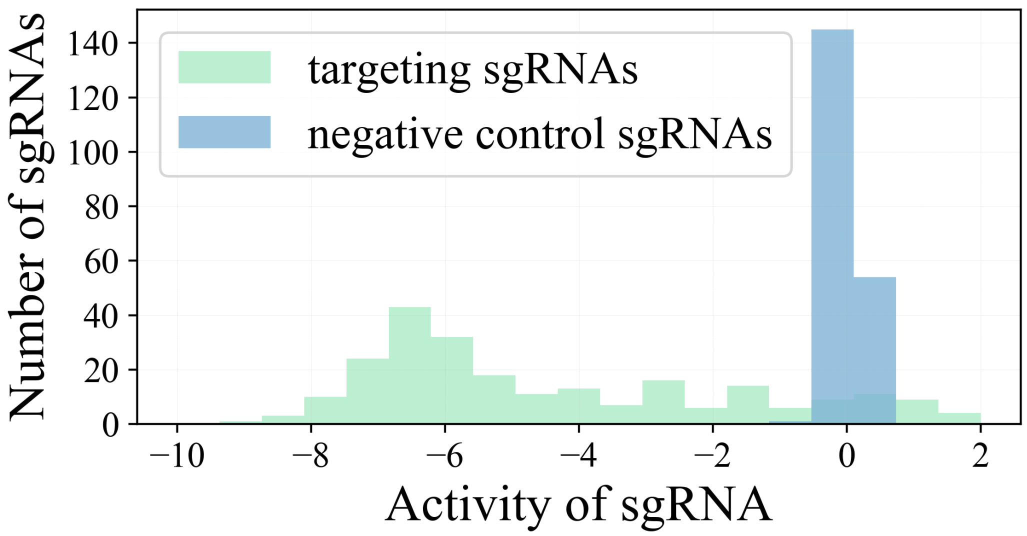

Supplementary Fig. 30 For the 16 false negative auxotrophic genes (see Supplementary Note I), their target activity scores (note that this value is the ability of sgRNA to cleave *E. coli* genomic DNA in the presence of Cas9, more negative, better activity) profiled in a recent work (Guo and Wang et al., BioRxiv: https://doi.org/10.1101/272377) are shown in green histogram in contrast to the blue negative control sgRNAs.

Supplementary Table 1 Targeted genes and relevant annotations in tiling library

Gene	Operon (RegulonDB)	Library	Description	Function
argA	*argA*	Library I	auxotrophic	amino acid *N*-acetyltransferase
argE	*argE*	Library I	auxotrophic	acetylornithine deacetylase
argG	*argG*	Library I	auxotrophic	argininosuccinate synthetase
bioH	*bioH*	Library I	auxotrophic	pimeloyl-ACP methyl ester carboxylesterase
cysB	*cysB*	Library I	auxotrophic	cysteine regulon transcriptional activator
cysE	*cysE*	Library I	auxotrophic	serine acetyltransferase
gltA	*gltA*	Library I	auxotrophic	citrate synthase
glyA	*glyA*	Library I	auxotrophic	serine hydroxymethyltransferase
icd	*icd*	Library I	auxotrophic	e14 prophage; isocitrate dehydrogenase
ilvC	*ilvC*	Library I	auxotrophic	ketol-acid reductoisomerase, NAD(P)-binding
lysA	*lysA*	Library I	auxotrophic	tagatose 6-phosphate aldolase 1, kbaZ subunit
metA	*metA*	Library I	auxotrophic	diaminopimelate decarboxylase, PLP-binding
panD	*panD*	Library I	auxotrophic	aminodeoxychorismate synthase, subunit II
panZ	*panZ*	Library I	auxotrophic	aspartate 1-decarboxylase
ppc	*ppc*	Library I	auxotrophic	phosphoenolpyruvate carboxylase
proC	*proC*	Library I	auxotrophic	pyrroline-5-carboxylate reductase, NAD(P)-binding
purC	*purC*	Library I	auxotrophic	phosphoribosylaminoimidazole-succinocarboxamide synthetase
purL	*purL*	Library I	auxotrophic	phosphoribosylformyl-glycineamide synthetase
purM	*purM*	Library I	auxotrophic	phosphoribosylaminoimidazole synthetase
pyrC	*pyrC*	Library I	auxotrophic	dihydro-orotase
pyrD	*pyrD*	Library I	auxotrophic	dihydro-orotate oxidase, FMN-linked
serA	*serA*	Library I	auxotrophic	D-3-phosphoglycerate dehydrogenase
agaV	*kbaZ_agaVWA*	Library II	auxotrophic	*N*-acetylgalactosamine-specific enzyme IIB component of PTS
aroF	*aroF_tyrA*	Library II	nonauxotrophic	3-deoxy-D-arabino-heptulosonate-7-phosphate synthase, tyrosine-repressible
cysG	*nirBDC_cysG*	Library II	auxotrophic	fused siroheme synthase
fic	*yhfG_fic_pabA*	Library II	nonauxotrophic	stationary-phase adenosine monophosphate-protein transferase domain protein
glnA	*glnALG*	Library II	auxotrophic	glutamine synthetase
glnG	*glnALG*	Library II	nonauxotrophic	fused DNA-binding regulator in two-component regulatory system with GlnL
glnL	*glnALG*	Library II	nonauxotrophic	sensory histidine kinase in two-component regulatory system with GlnG
gshB	*rsmE_gshB*	Library II	nonauxotrophic	glutathione synthetase
kbaZ	*kbaZ_agaVWA*	Library II	nonauxotrophic	transcriptional activator of ilvC; autorepressor
nadA	*nadA_pnuC*	Library II	auxotrophic	homoserine O-transsuccinylase
nadR	*serB_radA_nadR*	Library II	nonauxotrophic	quinolinate synthase, subunit A
nirB	*nirBDC_cysG*	Library II	nonauxotrophic	nicotinamide mononucleotide adenylyltransferase, ribosylnicotinamide kinase
nirC	*nirBDC_cysG*	Library II	nonauxotrophic	nitrite reductase, large subunit, NAD(P)H-binding
nirD	*nirBDC_cysG*	Library II	nonauxotrophic	nitrite transporter
pabA	*yhfG_fic_pabA*	Library II	auxotrophic	activator of AmiC murein hydrolase activity, lipoprotein
pnuC	*nadA_pnuC*	Library II	nonauxotrophic	nicotinamide riboside transporter
pyrB	*pyrLBI*	Library II	auxotrophic	aspartate carbamoyltransferase, catalytic subunit

pyrI	*pyrLBI*	Library II	nonauxotrophic	aspartate carbamoyltransferase, regulatory subunit
radA	*serB_radA_nadR*	Library II	nonauxotrophic	DNA repair protein
rsmE	*rsmE_gshB*	Library II	auxotrophic	16S rRNA m(3)U1498 methyltransferase, SAM-dependent
serB	*serB_radA_nadR*	Library II	auxotrophic	3-phosphoserine phosphatase
tyrA	*aroF_tyrA*	Library II	auxotrophic	fused chorismate mutase T/prephenate dehydrogenase

Supplementary Table 2 Quality metrics of NGS libraries constructed for the *E. coli* genome-wide sgRNA library screening experiments

Library	Pair-end read number	After pretreatment*	Mapping ratio&	Overall remaining ratio	Zero-count sgRNA	Gini Index
initial	18,041,548	0.793	0.839	0.666	479	0.1116
dCas9-R1	17,072,588	0.859	0.833	0.716	2,484	0.1792
dCas9-R2	16,251,128	0.800	0.831	0.665	2,916	0.1857
NC-R1	14,106,181	0.843	0.842	0.710	834	0.1111
NC-R2	19,063,793	0.812	0.841	0.683	856	0.1088
loginitial#	14,552,291	0.855	0.834	0.713	3,286	0.1842
LB-R1	19,629,340	0.856	0.833	0.712	4,057	0.2035
LB-R2	18,880,372	0.811	0.831	0.673	3,583	0.2056
MOPS-R1	14,420,545	0.867	0.833	0.722	4,270	0.2517
MOPS-R2	18,656,939	0.803	0.831	0.667	3,963	0.2486
MOPSC-R1	15,493,569	0.865	0.832	0.719	4,425	0.2244
MOPSC-R2	21,170,504	0.732	0.826	0.605	3,746	0.2203
MOPSF-R1	16,164,804	0.854	0.834	0.713	5,338	0.2885
MOPSF-R2	18,420,503	0.813	0.833	0.677	4,286	0.2816
MOPSI-R1	10,941,327	0.832	0.839	0.698	6,131	0.3165
MOPSI-R2	18,306,296	0.811	0.840	0.681	3,934	0.2516

* Pretreatment denotes removing those reads without corresponding pairs or poor quality, see Methods

& Mapping ratio of pretreated reads to the *in silico* library

Mixture of dCas9-R1 and dCas9-R2, see Methods and Supplementary Fig. 7

Supplementary Table 3 Upstream non-essential genes co-transcribed with essential genes (Keio collection, also with significant growth defect in CRISPRi screening, FDR < 0.05, fitness < 0) in the same polycistronic operons exhibit no growth phenotypes.

Non-essential genes with no growth phenotypes	Operon (red genes are Keio essential genes with significant growth defect)	Annotation of other upstream non-essential genes in the operon
ptsP	*rppH,ptsP,lgt,thyA*	The pehnotype of *rppH* is only moderate
nudC	*nudC,hemE,nfi*	
queA	*queA,tgt,yajC,secD*	The pehnotype of *tgt* is only moderate, while repression of *yajC* results in growth cessation
yicR	*yicR,rpmB,rpmG,mutM*	
wecA, wzzE, wecB, wecC, rffG, rffH, wecD, wecE, wzxE	*wecA,wzzE,wecB,wecC,rffG,rffH,wecD,wecE,wzxE,wzyE,wecG*	
nnr	*nnr,tsaE,amiB,mutL,miaA, hfq,hflX,hflK,hflC*	
rimJ, yceH, yceM	*rimJ,yceH,yceM,murJ*	
yjjB	*yjjB,dnaT,dnaC,yjjA*	Repression of *dnaT* results in growth cessation
damX, gph	*damX,dam,rpe,gph,trpS*	The pehnotypes of *dam* and *rpe* is only moderate
yffB	*yffB,dapE,ypfN*	
pabC	*pabC,yceG,tmk,holB,ycfH*	The pehnotype of *yceG* is only moderate
yoaA	*yoaA,tsaB,yeaY*	
gph	*aroK,aroB,damX,dam,rpe, gph,trpS*	The pehnotypes of *all genes upstream of* trpS are only moderate
mqsR	*mqsR,mqsA,ygiS*	
ychQ	*ychQ,ychA,kdsA*	The pehnotype of *ychA* is only moderate
ygiM	*ygiM,cca*	

Supplementary Table 4 Genes reported to be required by Keio collection but non-essential by CRISPRi screening

Gene	Fitness	*FDR*	Annotation	CRISPR/Cas9 knockout	number of sgRNAs	Gene length (bp)	Essentiality by Kato et al.
alsK	-0.221	0.356	D-allose kinase		15	930	non-essential
asd	-0.181	0.296	aspartate-semialdehyde dehydrogenase		15	1104	essential
bcsB	0.486	0.076	regulator of cellulose synthase		15	2340	non-essential
btuB	-0.284	0.270	vitamin B12/cobalamin transporter		15	1845	not detected
chpS	0.088	0.609	antitoxin of the ChpBS system	selected and succeed	9	252	non-essential
djlB	0.213	0.337	putative HscC co-chaperone		15	1428	not detected
entD	-0.121	0.572	phosphopantetheinyltransferase		14	621	non-essential
folK	0.101	0.454	pyrophosphokinase	selected and fail	15	480	essential
glmS	0.296	0.157	aminotransferase		15	1830	essential
gpsA	-0.121	0.406	glycerol-3-phosphate dehydrogenase	selected and succeed	15	1020	essential
grpE	-0.335	0.306	heat shock protein	selected and fail	15	594	essential
hemG	-0.391	0.090	protoporphyrin oxidase, flavoprotein		15	546	essential
mazE	0.230	0.292	antitoxin of the ChpA-ChpR system		10	249	non-essential
mlaB	-0.301	0.131	ABC transporter		9	294	non-essential
rsmI	-0.244	0.052	16S rRNA methyltransferase		15	861	non-essential
waaU	-0.037	0.748	lipopolysaccharide core biosynthesis		15	1074	non-essential
yagG	-0.119	0.476	CP4-6 prophage; putative sugar transporter		15	1383	non-essential
ydfB	-0.234	0.536	Qin prophage; uncharacterized protein		1	129	non-essential
ydiL	-0.371	0.053	putative HTH domain DNA-binding protein		15	357	non-essential
yefM	-0.192	0.447	antitoxin of the YoeB-YefM system		10	252	non-essential
yhhQ	-0.124	0.306	DUF165 family inner membrane protein	selected and succeed	15	666	non-essential
yiaD	-0.137	0.345	multicopy suppressor of bamB		15	660	not detected

Supplementary Table 5 ncRNA knockdowns found to confer growth defect in rich media and their annotations as well as relevant literatures.

gene	function	knockout growth phenotype	reference
spf	selective inhibition of translation	mild growth defect	Hatfull, G. F., et al., (1986). Journal of Bacteriology[1]
agrB	regulate the translation of toxin *dinQ*	lower growth rate	Weel-Sneve, R., et al., (2013). PLoS Genetics[2]
ssrA	release a stalled ribosome from 'broken' mRNA	growth defect	Komine, Y., et al., (1994). PNAS[3]
esrE	unknown	inviable#	Chen, Z., et al., (2012). FEBS Letters.[4]
rnpB	RNA processing	inviable	Waugh, D. S., et al., (1990). Journal of Bacteriology[5]
tff	involved in regulation of rpsB expression	unknown, downstream *rpsB* isessential	Aseev, L. V., et al., (2008). RNA[6]
ffs	component of the signal recognition particle (SRP)	inviable	Brown, S., et al., (1984). Journal of Molecular Biology[7]

Supplementary Table 6 Gene knockouts known to confer furfural or isobutanol tolerance identified in the CRISPRi screening experiments.

Furfural

gene	fitness in CRISPRi	FPR	strain	chemical	mutation in paper	reference
ppsR#	2.14	0.0107	*E. coli* K12 MG1655	multiple evolution paradigm, 0.5-1.5 g/L furfural, MOPS#	downregulation, TRMR	Glebes, T. Y. et al., (2014) *Biotech. and Bioeng.*
*yqhD**	1.16	0.0088	*E. coli* W strain	10-12.5 mM furfural, AM1 minimal medium	knockout	Wang, X. et al., (2013) *PNAS.&*

# also known as ydiA, enriched in three TRMR experiments

* Individual knockout was used to confirm the phenotype

& also reported in Miller E. N. et al., (2009) Appl. and Environ. Microbiol.

Isobutanol

gene	fitness in CRISPRi	FPR	strain	chemical	mutation in paper	reference
gatY	1.16	0.0017	*E. coli* BW25113	6 g/L isobutanol, LB broth*	insertion	Atsumi S. et al., (2010) *Molecular Systems Biology*
tnaA	0.68	0.0010	*E. coli* BW25113	6 g/L isobutanol, LB broth*	insertion	Atsumi S. et al., (2010) *Molecular Systems Biology*
yhbJ	0.83	0.0044	*E. coli* BW25113	6 g/L isobutanol, LB broth*	insertion	Atsumi S. et al., (2010) *Molecular Systems Biology*
marC&[***]	0.54	0.0073	*E. coli* BW25113	6 g/L isobutanol, LB broth*	deletion of marCRAB	Atsumi S. et al., (2010) *Molecular Systems Biology*

& No significant phenotype for *marRAB* in our dataset

*** *marC* knockout was also reported to have isobutanol tolerance phenotype by Minty J. J. et al., (2011) Microb. Cell. Fact.

Supplementary Table 7 Strains and plasmids used in this work

**Strain/plasmid**	**Characteristics**	**Source**
**Strains**		
E. coli K12 MG1655		ATCC 700926
E. coli s17-1	Expresses sfGFP protein	Ref. 1
E. coli MCm	Carries chloromycetin expression cassette integrated into *smf* locus of *E. coli* K12 MG1655	This study
E. coli Msac	Carries *sacB* expression cassette integrated into smf locus of *E. coli* K12 MG1655	This study
E. coli lyc001	Carries lycopene biosynthesis cluster (*crtEIB*) integrated into *ldhA* locus and key gene expression of MEP pathway modulated by promoter engineering in *E. coli* K12 MG1655	Unpublished data
**Plasmids**		
pKM154	sacB expression cassette, Amp^R, Cm^R	Addgene plasmid #13036
pTrc99a-*crt*-M	crtEIB gene cluster, Amp^R	Ref. 2
pdCas9-bacteria	dCas9 expression cassette, Cm^R, p15A	Addgene plasmid #44249
pCas	Cas9 expression cassette, used in CRISPR/Cas9-based recombineering (Msac construction), wild-type spCas9 promoter, Km^R	Addgene plasmid #62225
pdCas9-cons	pdCas9-bacteria with promoter and antibiotic resistance region replaced by those from pCas, p15A, Km^R	This study
pdCas9-J23109	pdCas9-cons with promoter replaced by constitutive Anderson promoter, J23109, p15A, Km^R	This study
pdCas9-J23111	pdCas9-cons with promoter replaced by constitutive Anderson promoter, J23111, p15A, Km^R	This study
pdCas9-J23112	pdCas9-cons with promoter replaced by constitutive Anderson promoter, J23112, p15A, Km^R	This study
pdCas9-J23113	pdCas9-cons with promoter replaced by constitutive Anderson promoter, J23113, p15A, Km^R	This study
pdCas9-J23116	pdCas9-cons with promoter replaced by constitutive Anderson promoter, J23116, p15A, Km^R	This study
pTargetF	sgRNA expression plasmid, pBR322, Spe^R	Addgene plasmid #62226
pTargetF_amp	sgRNA expression plasmid, pBR322, Amp^R	This study
pTargetF_*chpS*_140	sgRNA expression plasmid to introduce indel frameshift mutation in chpS gene, Amp^R	This study
pTargetF_*folK*_20	sgRNA expression plasmid to introduce indel frameshift mutation in *folK* gene, Amp^R	This study
pTargetF_*gpsA*_69	sgRNA expression plasmid to introduce indel frameshift mutation in *gpsA* gene, Amp^R	This study
pTargetF_*grpE*_40	sgRNA expression plasmid to introduce indel frameshift mutation in *grpE* gene, Amp^R	This study
pTargetF_*yhhQ*_196	sgRNA expression plasmid to introduce indel frameshift mutation in *yhhQ* gene, Amp^R	This study
pTargetF_smf2	sgRNA expression plasmid targeting *smf* used for CRISPR/Cas9-based recombineering (Msac construction), pBR322, Spe^R	This study
pTrc99a	Protein overexpression plasmid with lacI repressor expression cassette, pBR322, Amp^R	Lab stock

pTargetF_lac	Replaces sgRNA promoter of pTargetF with synthetic inducible version, $P_{LlacO-1}$, inserts *lacI* cassette from pTrc99a, replaces resistance marker with Amp^R cloned from pTrc99a with BsaI within ORF eliminated, pBR322, Amp^R	This study
pTargetF_lac_preLib	Replace the N20 region (between promoter and downstream Cas9 binding site) of pTargetF_lac with two BsaI restriction sites with opposite directions, which is used for sgRNA plasmid library construction by Golden Gate assembly, pBR322, Amp^R	This study
pTargetF_lac_*sfGFP*_36	Expresses sgRNA targeting *sfGFP* in *E. coli* s17-1, pBR322, Amp^R	This study
pTargetF_lac_*sfGFP*_83	Expresses sgRNA targeting *sfGFP* in *E. coli* s17-1, pBR322, Amp^R	This study
pTargetF_lac_*sfGFP*_166	Expresses sgRNA targeting *sfGFP* in *E. coli* s17-1, pBR322, Amp^R	This study
pTargetF_lac_*sfGFP*_257	Expresses sgRNA targeting *sfGFP* in *E. coli* s17-1, pBR322, Amp^R	This study
pTargetF_lac_*sfGFP*_414	Expresses sgRNA targeting *sfGFP* in *E. coli* s17-1, pBR322, Amp^R	This study
pTargetF_lac_*sfGFP*_631	Expresses sgRNA targeting *sfGFP* in *E. coli* s17-1, pBR322, Amp^R	This study
pTargetF_lac_*sacB*-1/2	Expresses sgRNA targeting *sacB* in *E. coli* MCm, pBR322, Amp^R	This study
pTargetF_lac_*crtE*-1/2	Expresses sgRNA targeting *crtE* in *E. coli* lyc001, pBR322, Amp^R	This study

Ref. 1: Lv L, Ren Y-L, Chen J-C, Wu Q, Chen G-Q. Metab Eng. 2015;29: 160–8.

Ref. 2: Zhao J, Li Q, Sun T, Zhu X, Xu H, Tang J, et al. Metab Eng. 2013;17: 42–50. doi:10.1016/j.ymben.2013.02.002

Supplementary Table 8 Primers and other oligonucleotides used in this work

Primer	Sequence	Usage
PF_smf_up	CGTGGCAGCAGTGAACCATGAACG	*smf* locus integration *(E. coli* MCm, *E. coli* Msac)
PR_smf_1	CCTTATCTCCCTGCCATAAGCAGCC	*smf* locus integration *(E. coli* MCm, *E. coli* Msac)
PF_smg_2	ATGCCATGTTCGACGTACTAATGTATTTGTTTG	*smf* locus integration *(E. coli* MCm, *E. coli* Msac)
PR_smg_down	GAAGTTAATGGCAAATTGACACTCCGGG	*smf* locus integration *(E. coli* MCm, *E. coli* Msac)
PF_CmCassette_HAsmf	GGCTGCTTATGGCAGGGAGATAAGGTCCGGGGATCCCGGACCGGGTCG	Cm^R cassette insertion into *smf* locus (*E. coli* MCm)
PR_CmCassette_HAsmf	TACATTAGTACGTCGAACATGGCATGTCTATTTTCCTCTTTTGTTTGATAGAAAATC	Cm^R cassette insertion into *smf* locus (*E. coli* MCm)
PF_105RBS-HAsmf	GGCTGCTTATGGCAGGGAGATAAGG	*sacB* cassette insertion into *smf* locus (*E. coli* Msac)
PR_105RBS-HAsacB	GTTTTGCAAACTTTTTGATGTTCATGGAC	*sacB* cassette insertion into *smf* locus (*E. coli* Msac)
PF_sacB	ATGAACATCAAAAAGTTTGCAAAACAAGCAAC	*sacB* cassette insertion into *smf* locus (*E. coli* Msac)
PR_sacBTer_smg	TACATTAGTACGTCGAACATGGCATCACGGCTGGGACGGAAGTCG	*sacB* cassette insertion into *smf* locus (*E. coli* Msac)
PF_Cas9ordCas9	ATGGATAAGAAATACTCAATAGGCTTAG	Clone dCas9+p15A from pdCas9-bacteria to construct pdCas9-cons
PR_Ter_p15A_HA	TTTGATGCTCGATGAGTTTTTCTAATAATTTGATATCGAGCTCGCTTGGACTCCTGTT	Clone dCas9+p15A from pdCas9-bacteria to construct pdCas9-cons
PF_neo_consPro	TTAGAAAAACTCATCGAGCATCAAATG	Clone constitutive promoter and Km^R from pCas9 to construct pdCas9-cons
PR_neo_consPro_HA	CTAAGCCTATTGAGTATTTCTTATCCAT	Clone constitutive promoter and Km^R from pCas9 to construct pdCas9-cons
Pdcas9-con-vetor-2-F	ATGGATAAGAAATACTCAATAGGCTTAGCTATCG	Construction of pdCas9_J23109/111/112/113/116
Pdcas9-con-vetor-2-R	CGCGCCAATGGACCAAC	Construction of pdCas9_J23109/111/112/113/116
promoter-F1	AGACACAACGTGGCTTTCCCTGCAG	Construction of pdCas9_J23109/111/112/113/116
promoter-F3	GGAGAAAGGATCTATGGATAAGAAATACTCAATAGGCT	Construction of pdCas9_J23109/111/112/113/116
Pj23109-F2	TTTACAGCTAGCTCAGTCCTAGGGACTGTGCTAGCGGCCGCAGATCTAAAGA	Construction of pdCas9_J23109/111/112/113/116
Pj23111-F2	TTGACGGCTAGCTCAGTCCTAGGTATAGTGCTAGCGGCCGCAGATCTAAAGA	Construction of pdCas9_J23109/111/112/113/116
Pj23112-F2	CTGATAGCTAGCTCAGTCCTAGGGATTATGCTAGCGGCCGCAGATCTAAAGA	Construction of pdCas9_J23109/111/112/113/116
Pj23113-F2	CTGATGGCTAGCTCAGTCCTAGGGATTATGCTAGCGGCCGCAGATCTAAAGA	Construction of pdCas9_J23109/111/112/113/116
Pj23116-F2	TTGACAGCTAGCTCAGTCCTAGGGACTATGCTAGCGGCCGCAGATCTAAAGA	Construction of pdCas9_J23109/111/112/113/116
promoter-R1	AGCCTATTGAGTATTTCTTATCCAT	Construction of pdCas9_J23109/111/112/113/116

promoter-R2	AGATCCTTTCTCCTCTTTAGATCTGCGGCC	Construction of pdCas9_J23109/111/112/113/116
Pj23109-R3	GCTAGCACAGTCCCTAGGACTGAGCTAGCTGTAAACTGCAGGGAAAGCCACGTTGTGTCT	Construction of pdCas9_J23109/111/112/113/116
Pj23111-R3	GCTAGCACTATACCTAGGACTGAGCTAGCCGTCAACTGCAGGGAAAGCCACGTTGTGTCT	Construction of pdCas9_J23109/111/112/113/116
Pj23112-R3	GCTAGCATAATCCCTAGGACTGAGCTAGCTATCAGCTGCAGGGAAAGCCACGTTGTGTCT	Construction of pdCas9_J23109/111/112/113/116
Pj23113-R3	GCTAGCATAATCCCTAGGACTGAGCTAGCCATCAGCTGCAGGGAAAGCCACGTTGTGTCT	Construction of pdCas9_J23109/111/112/113/116
Pj23116-R3	GCTAGCATAGTCCCTAGGACTGAGCTAGCTGTCAACTGCAGGGAAAGCCACGTTGTGTCT	Construction of pdCas9_J23109/111/112/113/116
seq-F1	ATGTAAGCAGACAGTTTTATTGTTCATG	Construction of pdCas9_J23109/111/112/113/116
seq-F2	GCGCTTAATCTATTTGGCCTTAGC	Construction of pdCas9_J23109/111/112/113/116
seq-R1	AACTTCTTCAAAATTCCATGGGG	Construction of pdCas9_J23109/111/112/113/116
PLlacO-F1	CTGGCAAATATTCTGAAATGAGCTGATAAATGTGAGCGGATAACATTGACATT	Construction of pTargetF_lac, $P_{LlacO-1}$ establishment
PLlacO-F2	GTGAGCGGATAACAAGATACTGAGCACAAACTCTGCACAACGTGATGGTTTT	Construction of pTargetF_lac, $P_{LlacO-1}$ establishment
PLlacO-R1	AAAACCATCACGTTGTGCAGAGTTT	Construction of pTargetF_lac, $P_{LlacO-1}$ establishment
PLlacO-R2	GTGCTCAGTATCTTGTTATCCGCTCACAATGTCAATGTTATCCGCTCACATTTAT	Construction of pTargetF_lac, $P_{LlacO-1}$ establishment
PLlacO-R3	CAGCTCATTTCAGAATATTTGCCAG	Construction of pTargetF_lac, $P_{LlacO-1}$ establishment
vector-F	AAACTCTGCACAACGTGATGGTTTTAG	Construction of pTargetF_lac, $P_{LlacO-1}$ establishment
vector-R	GGATCCAGCATATGCGGTGTG	Construction of pTargetF_lac, $P_{LlacO-1}$ establishment
lacI_ORF-F	ATTTCACACCGCATATGCTGGATCCGTGGTGAATGTGAAACCAGTAACGTTATAC	Construction of pTargetF_lac, *lacI* cassette clone
lacI_ORF-R	CAGCTCATTTCAGAATATTTGCCAGAAC	Construction of pTargetF_lac, *lacI* cassette clone
PF_BsaIcorrect	GAGCGTGGTTCTCGCGGTATC	Construction of pTargetF_lac, BsaI elimination
PR_BsaIcorrect	GATACCGCGAGAACCACGCTC	Construction of pTargetF_lac, BsaI elimination
PF_target_linearHA	TGCCTCACTGATTAAGCATTGGTAAATGCCGCTCGCCAGTCGATTG	Construction of pTargetF_lac, pTargetF_linearization
PR_target_linearHA	CGACACGGAAATGTTGAATACTCATGACATTGCACTCCACCGCTG	Construction of pTargetF_lac, pTargetF_linearization
PF_ampR	ATGAGTATTCAACATTTCCGTGTCG	Construction of pTargetF_lac, Amp^R cloning
PR_ampR	TTACCAATGCTTAATCAGTGAGGCAC	Construction of pTargetF_lac, Amp^R cloning
HS-BVC-B059	AATGCGCGCCATTACCGAGTC	Construction of pTargetF_lac_preLib
HS-BVC-B060	AACTGAGACCACAGGTCTCAGTGCTCAGTATCTTGTTATCCG	Construction of pTargetF_lac_preLib

HS-BVC-B061	TGAGACCTGTGGTCTCAGTTTTAGAGCTAGAAATAGCAAGTTAAAATAAGGCTAGTCCGTTATCAACTTGAAAAAGTGGCACCGAGTCG	Construction of pTargetF_lac_preLib
HS-BVC-B062	CTTCTGCAGGTCGACTCTAGAGAATTCAAAAAAAGCACCGACTCGGTGCCACTTTTTC	Construction of pTargetF_lac_preLib
PR_pTarget_toLacI	GGATCCAGCATATGCGGTGTG	Tuning of inducible sgRNA expression in pTargetF_lac
PF_pTarget_lacI	ATGGCGGAGCTGAATTACATTCC	Tuning of inducible sgRNA expression in pTargetF_lac
PR_pTarget-10Mut	CTCATTATATTGTTATCCGCTCACAATG	Tuning of inducible sgRNA expression in pTargetF_lac
PF_J23100_RBS	TTTCACACCGCATATGCTGGATCC	Tuning of inducible sgRNA expression in pTargetF_lac
PR_J23100_RBS	GGGAATGTAATTCAGCTCCGCCAT	Tuning of inducible sgRNA expression in pTargetF_lac
oligo_J23100F	TTTCACACCGCATATGCTGGATCCTTGACGGCTAGCTCAGTCCTAGGTACAGTGCTAGC	Tuning of inducible sgRNA expression in pTargetF_lac
oligo_J23100R	AGCTAGCCGTCAAGGATCCAGCATATGCGGTGTGAAA	Tuning of inducible sgRNA expression in pTargetF_lac
oligo_RBS0F	AGAAGGGATAAAAGTAACTATTAAGGAGGTAAATTATGGCGGAGCTGAATTACATTCCC	Tuning of inducible sgRNA expression in pTargetF_lac
oligo_RBS0R	AATTTACCTCCTTAATAGTTACTTTTATCCCTTCTGCTAGCACTGTACCTAGGACTG	Tuning of inducible sgRNA expression in pTargetF_lac
oligo_RBS1F	CCCTCCGGAAAAGAGGATCATAAGGACGGTAAAAAAATGGCGGAGCTGAATTACATTCCC	Tuning of inducible sgRNA expression in pTargetF_lac
oligo_RBS1R	TTTTTACCGTCCTTATGATCCTCTTTTCCGGAGGGGCTAGCACTGTACCTAGGACTG	Tuning of inducible sgRNA expression in pTargetF_lac
oligo_RBS2F	ACAATCAAAGAAGGAGATAAGGATAGAACAAGAATGGCGGAGCTGAATTACATTCCC	Tuning of inducible sgRNA expression in pTargetF_lac
oligo_RBS2R	TCTTGTTCTATCCTTATCTCCTTCTTTGATTGTGCTAGCACTGTACCTAGGACTG	Tuning of inducible sgRNA expression in pTargetF_lac
oligo_RBS3F	CTTCAGAGAATCAAATTAATAAGGACTTAAAATGGCGGAGCTGAATTACATTCCC	Tuning of inducible sgRNA expression in pTargetF_lac
oligo_RBS3R	TTTAAGTCCTTATTAATTTGATTCTCTGAAGGCTAGCACTGTACCTAGGACTG	Tuning of inducible sgRNA expression in pTargetF_lac
oligo_RBS4F	CAATCTAAACTATACACCGAAAAGACGAGTTCGAGATGGCGGAGCTGAATTACATTCCC	Tuning of inducible sgRNA expression in pTargetF_lac
oligo_RBS4R	CTCGAACTCGTCTTTTCGGTGTATAGTTTAGATTGGCTAGCACTGTACCTAGGACTG	Tuning of inducible sgRNA expression in pTargetF_lac
PF_smf_spc-2	TCCTAGGTATAATACTAGTGAGCTTTCGATACTCTTTCGGTTTTAGAGCTAGAAATAGC	Construction of pTargetF_smf2
PR_pTargetF_construct	ACTAGTATTATACCTAGGACTGAGCTAGCTGTCAAG	Construction of pTargetF_smf2
PR_pTargetF_lac_construct	GTGCTCAGTATCTTGTTATCCGC	Replace N20 region of pTargetF_lac plasmid
PF_sfGFP_36_spc	ATAACAAGATACTGAGCACCATCCAGTTCCACCAGAATAGTTTTAGAGCTAGAAATAGC	Replace N20 region of pTargetF_lac plasmid to construct pTargetF_lac_*sfGFP*_36
PF_sfGFP_83_spc	ATAACAAGATACTGAGCACACCTTCACCCTCGCCACGCAGTTTTAGAGCTAGAAATAGC	Replace N20 region of pTargetF_lac plasmid to construct pTargetF_lac_*sfGFP*_83

PF_sfGFP_166_spc	ATAACAAGATACTGAGCACGTCGTTACCAGAGTCGGCCAGTTTTAGAGCTAGAAATAGC	Replace N20 region of pTargetF_lac plasmid to construct pTargetF_lac_*sfGFP*_166
PF_sfGFP_257_spc	ATAACAAGATACTGAGCACCACATAGCCTTCCGGCATGGGTTTTAGAGCTAGAAATAGC	Replace N20 region of pTargetF_lac plasmid to construct pTargetF_lac_*sfGFP*_257
PF_sfGFP_414_spc	ATAACAAGATACTGAGCACTAAAATTGTATTCCAGCTTAGTTTTAGAGCTAGAAATAGC	Replace N20 region of pTargetF_lac plasmid to construct pTargetF_lac_*sfGFP*_414
PF_sfGFP_631_spc	ATAACAAGATACTGAGCACATATGATCGCGTTTCTCGTTGTTTTAGAGCTAGAAATAGC	Replace N20 region of pTargetF_lac plasmid to construct pTargetF_lac_*sfGFP*_631
PF_crtE_spc-1-m	ATAACAAGATACTGAGCACGTTGCTCAATGTCGTTCAACGTTTTAGAGCTAGAAATAGC	Replace N20 region of pTargetF_lac plasmid to construct pTargetF_lac_*crtE*-1
PF_crtE_spc-2-m	ATAACAAGATACTGAGCACAAGTCACGTTCGCTTTCAACGTTTTAGAGCTAGAAATAGC	Replace N20 region of pTargetF_lac plasmid to construct pTargetF_lac_*crtE*-2
PF_sacB_spc-1-m	ATAACAAGATACTGAGCACATGCCGTATGTTTCCTTATAGTTTTAGAGCTAGAAATAGC	Replace N20 region of pTargetF_lac plasmid to construct pTargetF_lac_*sacB*-1
PF_sacB_spc-2-m	ATAACAAGATACTGAGCACATTGTGGACGAATCGAATTCGTTTTAGAGCTAGAAATAGC	Replace N20 region of pTargetF_lac plasmid to construct pTargetF_lac_*sacB*-2
PF_pTargetF_chpS_140	TCCTAGGTATAATACTAGTTTCATCAAGCGAGTAGCGCCGTTTTAGAGCTAGAAATAGC	Replace N20 region of pTargetF_amp plasmid to construct pTargetF_*chpS*_140
oligo_chpS_140	GTTCATGTCACACTGTGCCAGCAGTTCATCAAGCGAGTAGCGCCTAGATGGGTGTCAGAATCAGTTGATTGTTGCTCACTTGCGCCTCCA	Oligonucleotide used during CRISPR/Cas9 recombineering to introduce indel mutation in *chpS*
PF_chpS_up	CGGGTGGCGTTGTTTTTTGC	Amplify the indel region of *chpS* for Sange sequencing
PR_chpS_down	CTTCGCAATGTAAAGGAACGC	Amplify the indel region of *chpS* for Sange sequencing
PF_pTargetF_folK_20	TCCTAGGTATAATACTAGTAGAGGCCAGATTGCTGCCTAGTTTTAGAGCTAGAAATAGC	Replace N20 region of pTargetF_amp plasmid to construct pTargetF_*folK*_20
oligo_folK_20	GCAAACGCGCACCACGTCGTGAGGGTACCGCATGACAGTGGCGTATATTGATAGGCAGCAATCTGGCCTCTCCGCTGGAGCAGGTCAATG	Oligonucleotide used during CRISPR/Cas9 recombineering to introduce indel mutation in *folK*
PF_folK_up	GACCTGTTGGCCTTGCGAG	Amplify the indel region of *folK* for Sange sequencing
PR_folK_down	GTTTAATTTGTCAAATGCTCTTGTATG	Amplify the indel region of *folK*for Sange sequencing
PF_pTargetF_gpsA_69	TCCTAGGTATAATACTAGTCCTCGTGGCCATTTCTTGCCGTTTTAGAGCTAGAAATAGC	Replace N20 region of pTargetF_amp plasmid to construct pTargetF_*gpsA*_69
oligo_gpsA_69	GGACAACCTCGTGGCCATTTCTTGCCAGTGATGGCAAGAGCGGTGCCGTACGAGCCGGCACCGATCACAGTCATTGAAGCATTACGTTGG	Oligonucleotide used during CRISPR/Cas9 recombineering to introduce indel mutation in *gpsA*
PF_gpsA_up	CGTATGCTCGTGAGTGCATC	Amplify the indel region of *gpsA* for Sange sequencing
PR_gpsA_down	CGCAGTGCAGCAGCTGCT	Amplify the indel region of *gpsA* for Sange sequencing

PF_pTargetF_grpE_40	TCCTAGGTATAATACTAGTTGATCCATGATAATTTCTTCGTTTTAGAGCTAGAAATAGC	Replace N20 region of pTargetF_amp plasmid to construct pTargetF_*grpE*_40
oligo_grpE_40	TCAACTGCCTCAATCTCTTCGTGCTGATCCATGATAATTTCTTCCGGCTTGCCCCTCAGGCGTTTTCTGTTCTTTACTACTCATGAATTT	Oligonucleotide used during CRISPR/Cas9 recombineering to introduce indel mutation in *grpE*
PF_grpE_up	TGCCAATACACTTGAAATGATTATTC	Amplify the indel region of *grpE* for Sange sequencing
PR_grpE_down	CCCTTCTGCATAATGCCCAG	Amplify the indel region of *grpE* for Sange sequencing
PF_pTargetF_yhhQ_196	TCCTAGGTATAATACTAGTAAGATAATGCGTCGGGCCAGGTTTTAGAGCTAGAAATAGC	Replace N20 region of pTargetF_amp plasmid to construct pTargetF_*yhhQ*_196
oligo_yhhQ_196	TTTTCTTGCTACCGACCTGACCGTGCGTATTTTTGGCGCAGCTGGCCCGACGCATTATCTTCGCGGTAATGATCCCTGCGTTATTAATCT	Oligonucleotide used during CRISPR/Cas9 recombineering to introduce indel mutation in *yhhQ*
PF_yhhQ_up	CCATTGCATTGTCAACGTAAAGC	Amplify the indel region of *yhhQ* for Sange sequencing
PR_yhhQ_down	CAGCGCGATTTCCATCCAG	Amplify the indel region of *yhhQ* for Sange sequencing
PF_pTargetLacNGS_SE75	ACATTGACATTGTGAGCG	PCR amplify N20 region of library plasmids for Illumina sequencing (SE75), tiling library
PR_pTargetLacNGS_SE75	ACTTGCTATTTCTAGCTC	PCR amplify N20 region of library plasmids for Illumina sequencing (SE75), tiling library
PF_pTargetLacNGS_PE150	TGCGCCGACATCATAACGGTTCTG	PCR amplify N20 region of library plasmids for Illumina sequencing (PE150), genome-wide library
PR_pTargetLacNGS_PE150	CGACTCGGTGCCACTTTTTCAAGTTG	PCR amplify N20 region of library plasmids for Illumina sequencing (PE150), genome-wide library

Underline suggests the N20 region within primers as the specificity guide for constructed sgRNAs

Supplementary Note 1 (false negative in auxotrophic gene screening)

We aimed to study the reason of false negative genes in auxotrophic experiment. The main purpose is to understand such failure is due to the poor sgRNA activity or experimental settings. Because the paper describing Keio collection does not give an exact definition for auxotrophic genes in MOPS medium, we hence checked the OD600 distribution of Keio collection in MOPS medium (Supplementary Fig. 28) and chose $OD_{600}<0.09$ as threshold to define auxotroph.

Meanwhile, FDR > 0.05 or median Z score > 0 is used to define gene knockdown without significant growth impairment in MOPS medium. Combining these two set together, we identified 16 false negative genes in genome-wide CRISPRi screening for auxotroph. The distribution of sgRNA fitness exhibits unimodal distribution (with average around 0), the same as genes that work (see Supplementary Fig. 29 for representative examples, *trpABC* is used as true positive example).

We think this phenomenon is not related to poor sgRNA activities. As observed in Supplementary Fig. 10, more than 90% sgRNAs in the library should be highly active. Moreover, according to our recent work using Cas9 (rather than dCas9 in this work) to probe sgRNA activities in the same *E. coli* host (Guo and Wang et al., BioRxiv: https://doi.org/10.1101/272377), the majority of sgRNAs belonging to these 16 genes presents strong activities (Supplementary Fig. 30). Although we cannot simply regard sgRNA activity of CRISPRi and CRISPR/Cas9 DNA cleavage as the same, this result still suggests indirectly that these sites are accessible for CRISPR/(d)Cas9 complex, a necessary condition for CRISPRi to work.

Hence, it is reasonable to suggest that targeting these 16 genes with any sgRNA does not cause a phenotype in our screening experiments. It can be deduced to the pooled

format of CRISPRi screening, where the cross-feeding of essential nutrient is possible, which is demonstrated in our previous paper (BioRxiv: doi: https://doi.org/10.1101/129668). The cell lysis upon repressing some genes may further makes this problem more severe. Another possibility is that the stock of nutrient (such as cofactor biotin) in the cell from seed culture is enough for cells to growth for 5 doublings without any supplementation.

Supplementary Methods

Statistical information and software used in this work

Plots were generated in Python 2.7 using the matplotlib (2.0.2) plotting libraries. Genome plots were generated using the Circos software package[8]. GO enrichment analysis was performed with the GOATOOLS Python package[9]. All statistical analyses, data fitting, interpolation calculations and machine learning were performed using the SciPy (0.19.1), NumPy (1.13.1) and scikit-learn (0.19.0) Python packages.

Supplementary references

1. Hatfull, G. F. & Joyce, C. M. Deletion of the spf (spot 42 RNA) gene of Escherichia coli. *J. Bacteriol.* **166,** 746–50 (1986).
2. Weel-Sneve, R. *et al.* Single Transmembrane Peptide DinQ Modulates Membrane-Dependent Activities. *PLoS Genet.* **9,** e1003260 (2013).
3. Komine, Y., Kitabatake, M., Yokogawa, T., Nishikawa, K. & Inokuchi, H. A tRNA-like structure is present in 10Sa RNA, a small stable RNA from Escherichia coli. *Proc. Natl. Acad. Sci. U. S. A.* **91,** 9223–7 (1994).
4. Chen, Z. *et al. Esre* : A novel essential non-coding RNA in *Escherichia coli. FEBS Lett.* **586,** 1195–1200 (2012).
5. Waugh, D. S. & Pace, N. R. Complementation of an RNase P RNA (rnpB) gene deletion in Escherichia coli by homologous genes from distantly related eubacteria. *J. Bacteriol.* **172,** 6316–22 (1990).
6. Aseev, L. V., Levandovskaya, A. A., Tchufistova, L. S., Scaptsova, N. V. & Boni, I. V. A new regulatory circuit in ribosomal protein operons: S2-mediated control of the rpsB-tsf expression in vivo. *RNA* **14,** 1882–1894 (2008).
7. Brown, S. & Fournier, M. J. The 4.5 S RNA gene of Escherichia coli is essential for cell growth. *J. Mol. Biol.* **178,** 533–50 (1984).
8. Krzywinski, M. *et al.* Circos: An information aesthetic for comparative genomics. *Genome Res.* **19,** 1639–1645 (2009).
9. Tang, H. *et al.* GOATOOLS: Tools for Gene Ontology. *Zenodo* (2015). doi:10.5281/ZENODO.31628
