# Supplementary material for: Pooled CRISPR interference screening enables genome-scale functional genomics study in bacteria with superior performance
Source: Nat Commun. 2018 Jun 26;9:2475. doi: 10.1038/s41467-018-04899-x (PMC6018678; doi:10.1038/s41467-018-04899-x)
Supplement: Supplementary file 3 — Description of Additional Supplementary Files [file 41467_2018_4899_MOESM3_ESM.pdf]

Description of Additional Supplementary Files

File Name: Supplementary Data 1
Description: In silico tiling sgRNA library.

File Name: Supplementary Data 2
Description: Gene clusters in genome-scale sgRNA library

File Name: Supplementary Data 3
Description: E. coli genome-wide sgRNA library

File Name: Supplementary Data 4
Description: Number of sgRNAs per gene of genome-wide library

File Name: Supplementary Data 5
Description: Gene fitness scores for genome-wide library screenings

File Name: Supplementary Data 6
Description: Fitness scores for sgRNAs of essential gene

File Name: Supplementary Data 7
Description: Fitness scores for sgRNAs of auxotrophy

File Name: Supplementary Data 8
Description: Fitness scores for sgRNAs of amino acid addition

File Name: Supplementary Data 9
Description: Fitness scores for sgRNAs of furfural addition

File Name: Supplementary Data 10
Description: Fitness scores for sgRNAs of isobutanol addition

File Name: Supplementary Data 11
Description: Essential gene set reported by Keio collection

File Name: Supplementary Data 12
Description: Genome-wide sgRNA library for model prokaryotic microorganisms

File Name: Supplementary Data 13
Description: Gene level metrics of genome-wide sgRNA library for model microorganisms

File Name: Supplementary Data 14
Description: Sublibrary composition of E. coli genome-scale sgRNA library
